# Commensal *Akkermansia muciniphila* Exacerbates Gut Inflammation in *Salmonella* Typhimurium-Infected Gnotobiotic Mice

**DOI:** 10.1371/journal.pone.0074963

**Published:** 2013-09-10

**Authors:** Bhanu Priya Ganesh, Robert Klopfleisch, Gunnar Loh, Michael Blaut

**Affiliations:** 1 Department of Gastrointestinal Microbiology, German Institute of Human Nutrition Potsdam-Rehbrücke, Nuthetal, Germany; 2 Institute of Veterinary Pathology, Free University Berlin, Berlin, Germany; French National Centre for Scientific Research, France

## Abstract

Excessive mucin degradation by intestinal bacteria may contribute to inflammatory bowel diseases because access of luminal antigens to the intestinal immune system is facilitated. This study investigated how the presence of a mucin degrading commensal bacterium affects the severity of an intestinal *Salmonella enterica* Typhimurium-induced gut inflammation. Using a gnotobiotic C3H mouse model with a background microbiota of eight bacterial species (SIHUMI) the impact of the mucin-degrading commensal bacterium *Akkermansia muciniphila* (SIHUMI-A) on inflammatory and infectious symptoms caused by *S.* Typhimurium was investigated. Presence of *A. muciniphila* in *S.* Typhimurium-infected SIHUMI mice caused significantly increased histopathology scores and elevated mRNA levels of IFN-γ, IP-10, TNF-α, IL-12, IL-17 and IL-6 in cecal and colonic tissue. The increase in pro-inflammatory cytokines was accompanied by 10-fold higher *S.* Typhimurium cell numbers in mesenteric lymph nodes of SIHUMI mice associated with *A. muciniphila* and *S.* Typhimurium (SIHUMI-AS) compared to SIHUMI mice with *S.* Typhimurium only (SIHUMI-S). The number of mucin filled goblet cells was 2- to 3- fold lower in cecal tissue of SIHUMI-AS mice compared to SIHUMI-S, SIHUMI-A or SIHUMI mice. Reduced goblet cell numbers significantly correlated with increased IFN-γ mRNA levels (r^2^ = −0.86, ***P<0.001) in all infected mice. In addition, loss of cecal mucin sulphation was observed in SIHUMI mice containing both *A. muciniphila* and *S.* Typhimurium compared to other mouse groups. Concomitant presence of *A. muciniphila* and *S.* Typhimurium resulted in a drastic change in microbiota composition of SIHUMI mice: the proportion of *B. thetaiotaomicron* in SIHUMI-AS mice was 0.02% of total bacteria compared to 78% – 88% in the other mouse groups and the proportion of *S.* Typhimurium was 94% in SIHUMI-AS mice but only 2.2% in the SIHUMI-S mice. These results indicate that *A. muciniphila* exacerbates *S.* Typhimurium-induced intestinal inflammation by its ability to disturb host mucus homeostasis.

## Introduction

The intestinal mucus layer provides a barrier against invasion of the epithelium by intestinal bacteria. Recent studies suggest that the pathophysiology of ulcerative colitis (UC) involves a disruption of the mucus layer integrity followed by depletion of mucus secretory goblet cells [1]–[4]. Besides serving as a barrier, mucus also represents a growth substrate and a site of adhesion for intestinal bacteria [2], [5]. Excessive mucin degradation by intestinal bacteria may contribute to inflammatory bowel diseases (IBD) by facilitating the access of luminal antigens to the intestinal immune system and by changes in the resident gut microbial community [6]–[12].

Using IL-10^−/−^ mice as a model of chronic gut inflammation, we previously observed that intestinal inflammation was reduced after 8 weeks of treatment with the probiotic bacterium *Enterococcus faecium* NCIMB 10415. This reduction in inflammation coincided with a lower abundance of *Akkermansia muciniphila*, a mucin-degrading commensal and a member of the Verrucomicrobia, from 10^8^ to 10^4^ cells g^−1^, suggesting that this organism promoted inflammation [13]. Moreover, in a T-cell transfer-mediated mouse model of intestinal inflammation the proportion of bacteria belonging to the phylum Verrucomicrobia was fivefold increased compared to control mice [14]. *A. muciniphila* is the main intestinal representative of this phylum [2], suggesting that *A. muciniphila* numbers increased in response to inflammation [14]. *A. muciniphila* is a commensal bacterium that colonizes the human gut early in life [15]–[17]. Because of its ability to degrade mucins, we hypothesized that this organism might contribute to intestinal inflammation.

To test this hypothesis we took advantage of a well-defined gnotobiotic mouse model associated with a defined simplified human intestinal microbiota (SIHUMI) of eight bacterial species [18], complemented with *A. muciniphila* or/and with *Salmonella enterica* Serovar Typhimurium (*S.* Typhimurium). The latter is a murine pathogen [19] that triggers acute inflammatory responses [20] in TLR11 knock-out mice or streptomycin-treated mice [21], [22] and therefore represents a highly suitable model for investigating immune disorders [19]. We therefore used *Salmonella* Typhimurium to induce intestinal inflammation in SIHUMI mice to investigate whether *A. muciniphila* influences the infectious and inflammatory symptoms caused by *Salmonella* Typhimurium in these mice. Here we demonstrate that *A. muciniphila* exacerbates *S.* Typhimurium-induced inflammation in the SIHUMI mouse model indicating that the former organism turns into a harmful bacterium under inflammatory conditions. Our experiments suggest that this is at least in part based on *A. muciniphila*'s ability to interfere with host mucus formation and production.

## Results

### *A. muciniphila* profoundly affects microbial community composition of SIHUMI mice associated with *S.* Typhimurium

**Figure 1:**
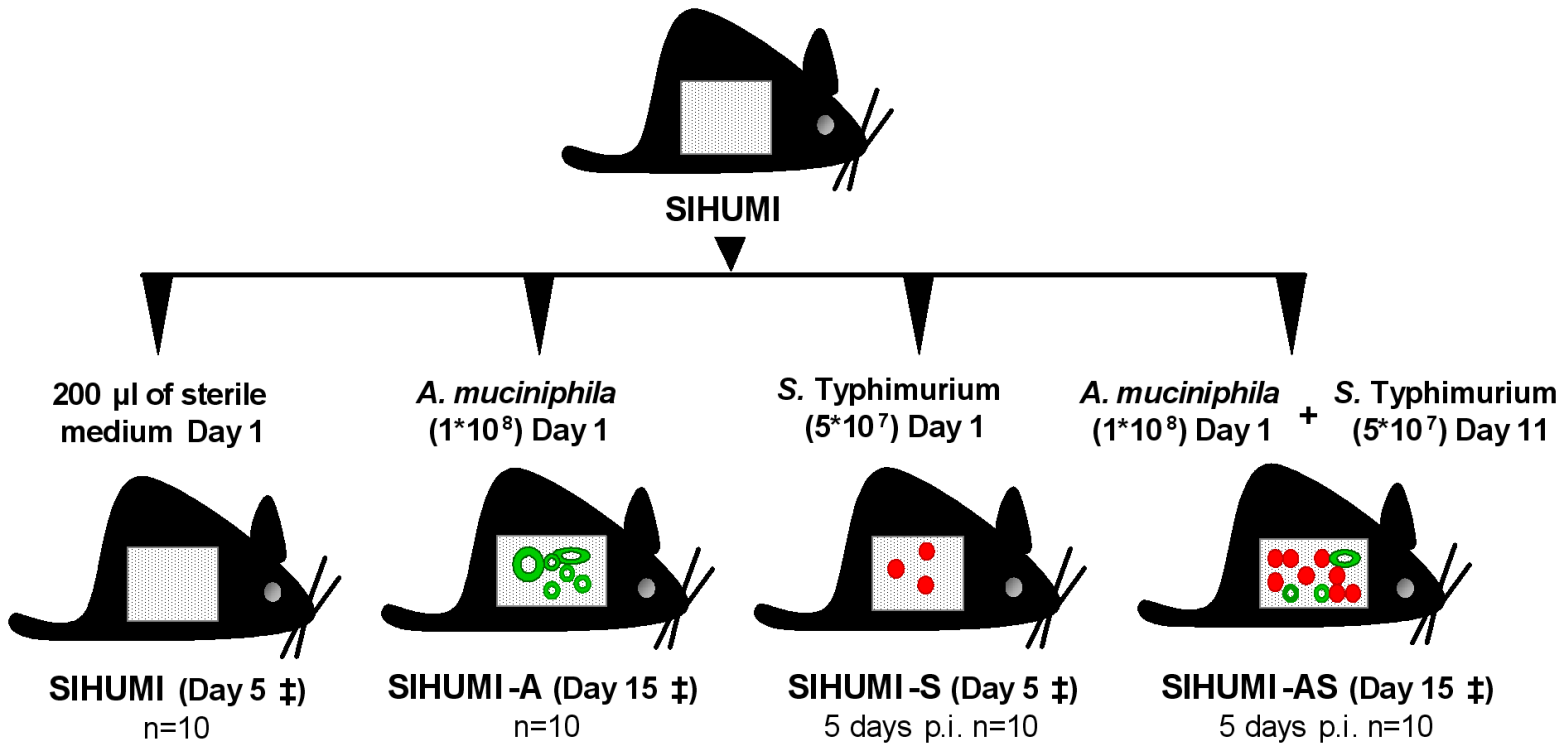
Design of the animal experiment. Fourty C3H mice associated with a defined microbial community of 8 bacterial species (SIHUMI) were allocated to four different groups (10 mice per group). Each mouse was associated with 8 bacterial species (SIHUMI). Twelve weeks-old SIHUMI mice were subsequently associated with *A. muciniphila* (SIHUMI-A) or *S.* Typhimurium (SIHUMI-S) or with both *A. muciniphila* and *S.* Typhimurium (SIHUMI-AS). SIHUMI mice received only sterile medium. Times of association, infection and killing are as indicated. ‡ - killed.

**Figure 2:**
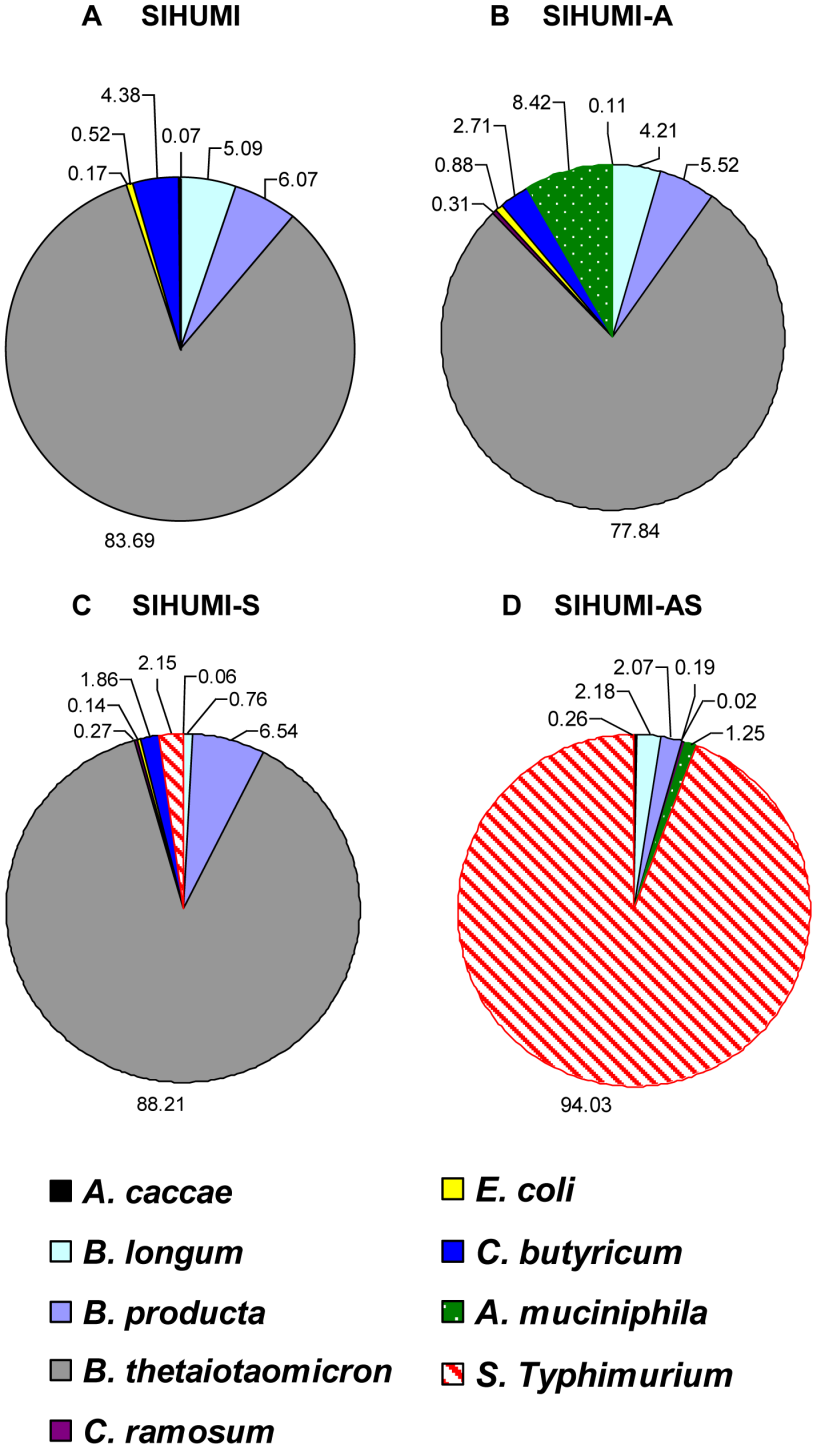
Presence of *A. muciniphila* renders *S.* Typhimurium the dominant species in gnotobiotic SIHUMI mice. Cecal contents were collected from gnotobiotic C3H mice, differing in their microbial status: (A) Mice with a defined microbial community of eight bacterial species (SIHUMI), (B) SIHUMI mice additionally colonized with *A. muciniphila* (SIHUMI-A), (C) SIHUMI mice infected with *S.* Typhimurium (SIHUMI-S) and (D) SIHUMI mice colonized with *A. muciniphila* and 10 days later infected with *S.* Typhimurium (SIHUMI-AS) (see Figure 1). Total DNA was extracted and bacterial cell numbers were quantified by qPCR with primers targeting the HSP60 gene of the SIHUMI members, the 16S rRNA gene of *A. muciniphila* and the ttr-region of *S.* Typhimurium. Calculation of the cell numbers was based on DNA obtained from cell suspensions containing known cell numbers of the targeted bacterial species (see materials and methods). Presence of *A. muciniphila* in SIHUMI-AS mice is attributed to an increase in the proportion of *S.* Typhimurium cells at the expense of other community members showing reduced proportion of SIHUMI members. Ten animals per group were used. The bacterial cell numbers and *P-values* for the differences between the groups are provided in Table 1.

To induce intestinal inflammation, mice associated with a simplified intestinal microbiota (SIHUMI) were additionally colonized with *A. muciniphila* and subsequently infected with *S.* Typhimurium (SIHUMI-AS). SIHUMI mice and SIHUMI mice associated with either *A. muciniphila* (SIHUMI-A) or *S.* Typhimurium (SIHUMI-S) served as controls (Figure 1). Bacterial cell numbers in the intestinal contents were quantified using qPCR (Figure 2). Five days post infection (p.i.) *S.* Typhimurium became the predominant species representing 94% of total bacteria in the cecum of SIHUMI-AS mice. In contrast, in SIHUMI-S mice *S.* Typhimurium made up merely 2.2% of total bacteria. *A. muciniphila* accounted for 8.4% of total bacterial cells in the SIHUMI-A group, but was as low as 1.3% in the SIHUMI-AS group. *B. thetaiotaomicron* was dominant in SIHUMI, SIHUMI-A and SIHUMI-S mice making up 80–90% of total bacteria but was reduced to 0.02% in the SIHUMI-AS mice. The proportion of other community members was also lower in the SIHUMI-AS group compared to the other mouse groups. For example, *E. coli* became undetectable in the SIHUMI-AS mice whereas this organism made up 0.14% of total bacteria in the SIHUMI-S animals. This was less than the initial *E. coli* proportion of 0.52% and 0.88% in the uninfected control groups SIHUMI and SIHUMI-A, respectively. Interestingly, there was no significant difference in the absolute *S.* Typhimurium cell numbers between SIHUMI-S and SIHUMI-AS mice but all other members of the community were 1 to 5 logs lower when both *S.* Typhimurium and *A. muciniphila* were present (SIHUMI-AS) suggesting that the latter organism caused a decrease of all other community members except *S.* Typhimurium (Table 1). Bacterial cell numbers in the colon revealed a pattern similar to that observed for cecum (Figure S1 and Table S1).

**Table 1:** *S.* Typhimurium becomes the dominant species in SIHUMI mice previously associated with *A. muciniphila*.

Cecum	SIHUMI	SIHUMI-A	SIHUMI-S	SIHUMI-AS
	log _10_ (g^−1^ DW)	log _10_ (g^−1^ DW)	log _10_ (g^−1^ DW)	log _10_ (g^−1^ DW)
*A. caccae*	8.20±0.26 *^ab^*	8.52±0.21 *^b^*	8.15±0.31 *^ab^*	7.45±1.26 *^a^*
*B. longum*	10.03±0.44 *^b^*	10.11±0.27 *^b^*	9.22±0.55 *^ab^*	8.37±2.10 *^a^*
*B. product*	10.11±0.35 *^b^*	10.23±0.29 *^b^*	10.16±0.26 *^b^*	8.34±1.34 *^a^*
*B. thetaiotaomicron*	11.25±0.20 *^b^*	11.38±0.22 *^b^*	11.29±0.30 *^b^*	6.29±0.75 *^a^*
*C. ramosum*	8.55±0.27 *^a^*	8.98±0.37 *^a^*	8.76±0.28 *^a^*	7.31±0.98 *^a^*
*E. coli*	9.05±0.19 *^bc^*	9.43±0.98 *^c^*	8.48±0.28 *^bc^*	4.90±0.41 *^a^*
*C. butyricum*	9.97±0.25 *^b^*	9.92±0.34 *^b^*	9.61±0.35 *^b^*	4.15±0.50 *^a^*
*A. muciniphila*	n.d.	10.41±0.20 *^b^*	n.d.	8.12±0.60 *^a^*
*S.* Typhimurium	n.d.	n.d.	9.67±0.16 *^a^*	9.99±0.45 *^a^*
Total bacteria	11.99±0.54 *^bc^*	11.65±0.62 *^b^*	11.96±0.43 *^bc^*	10.62±1.02 *^a^*

Data are expressed as mean±standard error. Different superscripts indicate statistically significant differences (*P≤0.05).* n = 10 mice per group. DW: dry weight.

### Presence of *A. muciniphila* aggravates inflammatory symptoms caused by *S.* Typhimurium in SIHUMI mice

**Figure 3:**
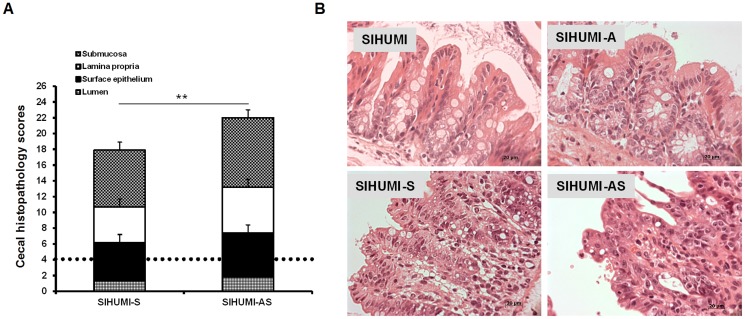
Concomitant presence of *A. muciniphila* and *S.* Typhimurium results in increased histopathology scores in SIHUMI mice. (A) Gnotobiotic C3H mice containing 8 defined microbial species (SIHUMI) were subsequently inoculated with *A. muciniphila* or *S.* Typhimurium or consecutively with both organisms (see Figure 1). SIHUMI and SIHUMI-A mice had the lowest histopathology scores (≤4.0) with no signs of inflammation and were therefore taken as baseline (dotted line). Data are expressed as median with range. *P<0.05, **P<0.01, ***P<0.001. n = 10 mice per group. (B) Representative microscopy images of pathological changes observed in cecum tissue sections fixed with formalin and stained with hematoxylin and eosin (4 µm) of the four mouse groups. n = 10 mice per group; Magnification: 1000-fold.

Histopathological analysis revealed that 5 days p.i. SIHUMI-AS mice showed a 24% higher cecal histopathology score compared to the SIHUMI-S mice and more than 4.5- to 5-fold higher scores compared to the SIHUMI and the SIHUMI-A mice (Figure 3). This result indicates that *A. muciniphila* exacerbates the symptoms of cecal inflammation caused by *S.* Typhimurium infection in SIHUMI-AS mice. The colon of the infected mice did not display histopathological signs of inflammation (data not shown).

**Figure 4:**
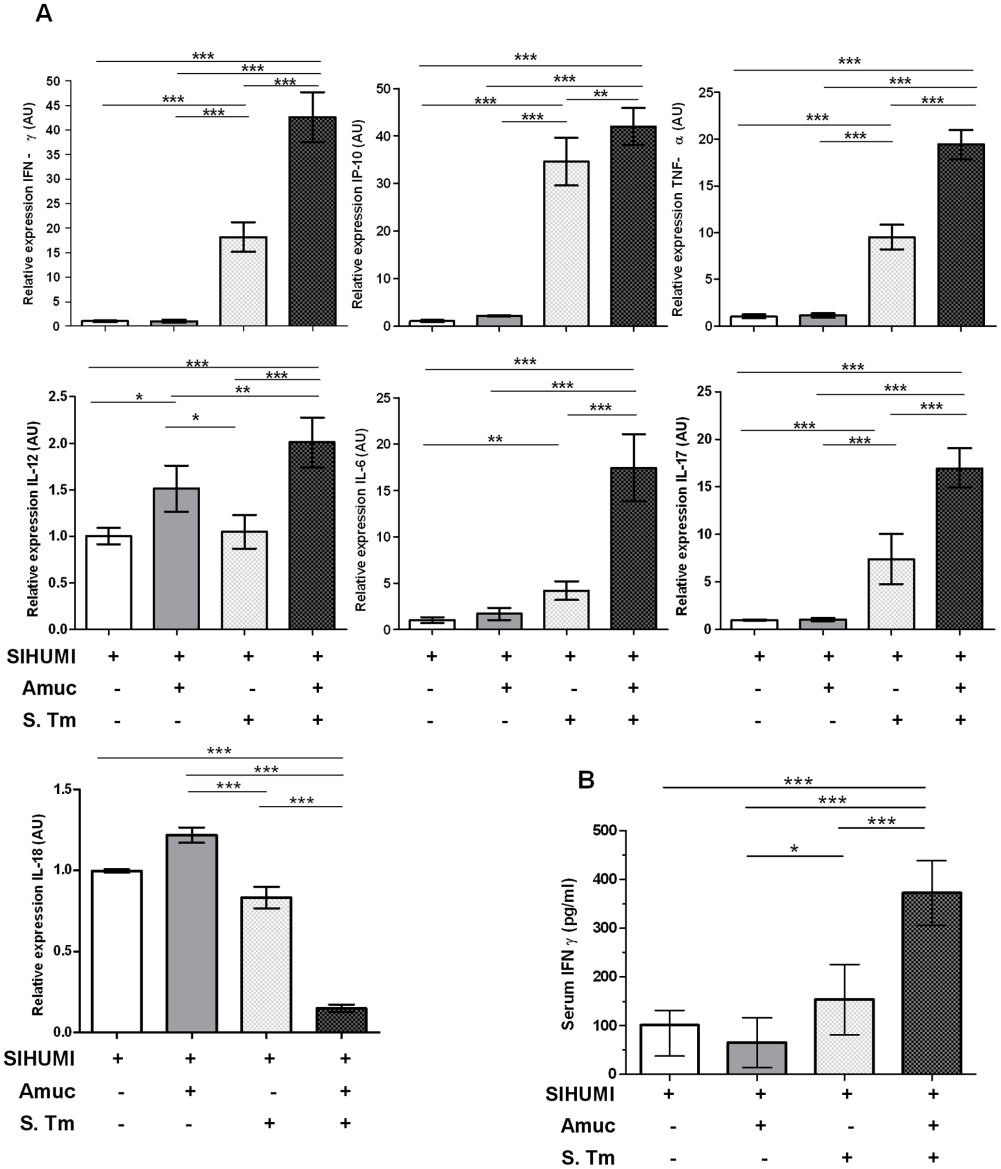
Presence of both *A. muciniphila* and *S.* Typhimurium is accompanied by increased pro-inflammatory cytokines. (A) Cecal mRNA levels of IFN-γ, IP-10, TNF-α, IL-12, IL-6, IL-17 and IL-18 in gnotobiotic SIHUMI mice were measured. mRNA was extracted from cecum mucosa of mice belonging to either one of four groups: SIHUMI, SIHUMI-A, SIHUMI-S and SIHUMI-AS (see Figure. 1). The mRNA was converted to cDNA for quantitative real-time PCR measurement (see materials and methods). Inoculation of the gnotobiotic SIHUMI mice with *A. muciniphila* followed by *S.* Typhimurium infection (SIHUMI-AS) caused an increase in mRNA levels of pro-inflammatory cytokines except IL-18. Data are expressed as mean±standard error. n = 6 per group. Star indicates statistically significant differences (*P<0.05, **P<0.01, ***P<0.001). AU: Arbitrary units; Amuc: *A. muciniphila;* S. Tm: *S.* Typhimurium. (B) Serum protein levels of IFN-γ were increased in SIHUMI-AS mice compared to the other mouse groups. Data are expressed as mean±standard error. n = 10 mice per group. *P<0.05, **P<0.01, ***P<0.001. Amuc: *A. muciniphila;* S. Tm: *S.* Typhimurium.

In line with the histopathology scores, mRNA levels of selected pro-inflammatory cytokines in cecal mucosa were up-regulated in the SIHUMI-AS mice 5 days p.i. compared to all other groups (Figure 4A). Interferon-gamma (IFN-γ) expression was approximately 2.5-fold higher in SIHUMI-AS mice compared to SIHUMI-S mice and approximately 40-fold higher compared to SIHUMI or SIHUMI-A mice. Essentially similar patterns were observed for IFN-γ-induced protein 10 (IP-10), tumor necrosis factor-α (TNF-α), interleukin (IL)-6 and IL-17. The pattern for IL-12 differed from that of the other cytokines investigated, with 1.5- and 2- fold higher IL-12 mRNA levels in the SIHUMI-A and SIHUMI-AS groups compared to the other two groups. In spite of these minor differences, the presence of *A. muciniphila* in the *S.* Typhimurium-infected SIHUMI mice coincided with significantly higher mRNA expression levels of the pro-inflammatory cytokines except IL-18, which was significantly down-regulated. The mRNA expression patterns of IFN-γ, IL-17, IL-6, TNF-α, IL-12 and IP-10 in colonic tissue were very similar to those observed in cecal tissue (Figure S2).

To check for systemic effects of infection, we also quantified the protein levels of pro-inflammatory cytokines in serum (Figure 4B). Five days p.i. SIHUMI-AS mice had 1.5- to 3- fold higher serum levels of IFN-γ compared to SIHUMI-S mice, SIHUMI mice or SIHUMI-A mice. However, TNF-α and IL-6 protein levels in serum were below the detection limit.

**Figure 5:**
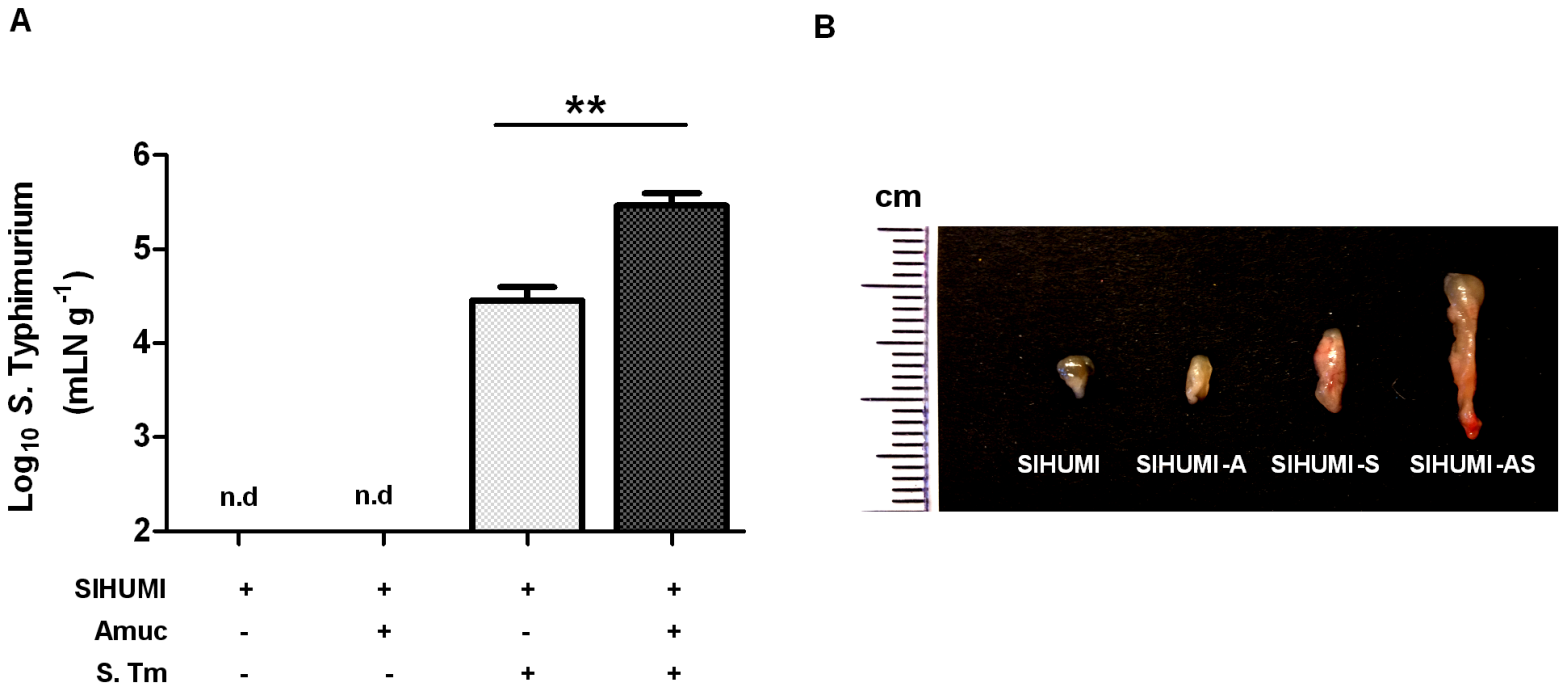
SIHUMI mice colonized with both *A. muciniphila* and *S.* Typhimurium display enlarged mLN and elevated *S.* Typhimurium cell numbers. (A) Mesenteric lymph nodes (mLN) were obtained from four groups of gnotobiotic C3H mice. SIHUMI mice were subsequently inoculated with *A. muciniphila* or *S.* Typhimurium or consecutively with both organisms (see Figure 1). The mLN tissue was homogenized and DNA was isolated to quantify *S.* Typhimurium using quantitative PCR with primers targeting the ttr-region of *S.* Typhimurium. Absolute cell numbers were calculated based on calibration curves with known concentrations of *S.* Typhimurium. The mLN of SIHUMI-AS mice contained 10-fold higher cell numbers of *S.* Typhimurium compared to SIHUMI-S mice. Data are expressed as mean±standard error. n = 10 mice per group. *P<0.05, **P<0.01, ***P<0.001. n.d: not detected. Amuc: *A. muciniphila;* S. Tm: *S.* Typhimurium. (B) The photograph shows four lymph nodes, each representative of one of the four mouse groups and a cm scale. Twelve week old gnotobiotic SIHUMI mice with both *A. muciniphila* and *S.* Typhimurium displayed an increased size of their mesenteric lymph nodes compared to SIHUMI mice infected with *S.* Typhimurium only.

The increased intestinal inflammation in the SIHUMI-AS mice compared to the SIHUMI-S mice coincided with a predominance of *S.* Typhimurium cells in the SIHUMI-AS mice suggesting that *A. muciniphila* exacerbated the pathogen-induced inflammation. To investigate whether the increased inflammation was accompanied by an enhanced translocation of *S.* Typhimurium into host tissue, *S.* Typhimurium was enumerated in mesenteric lymph nodes (mLN) and spleen. Five days p.i. the cell number of *S.* Typhimurium in the mLN of SIHUMI-AS mice was 10-fold higher compared to that observed for SIHUMI-S mice (Figure 5). However, *S.* Typhimurium was not detectable in the spleens of the mice infected with the pathogen.

**Figure 6:**
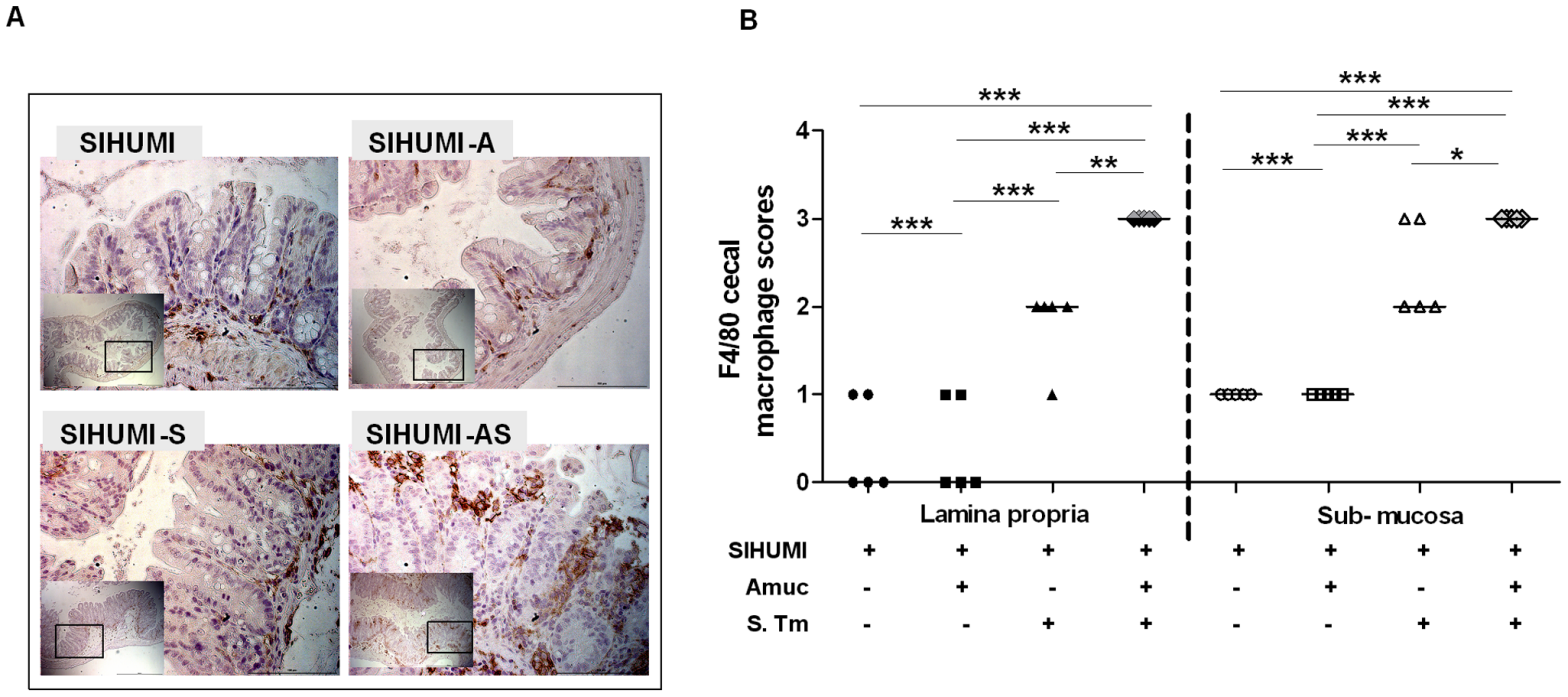
SIHUMI mice colonized with both *A. muciniphila* and *S.* Typhimurium display an increased cecal macrophage infiltration. (A) Formalin fixed paraffin embedded cecum tissue was thin sectioned at 2 µm. Macrophages were stained by targeting the F4/80 receptor expressed on mouse macrophages using immunohistochemistry with specific antibodies. Brown color indicates positively stained macrophages. Magnification 400-fold. Bar = 100 µm. (B) Positively stained macrophages were enumerated along a stretch of 50 µm of lamina muscularis for both lamina propria and sub-mucosa (see materials and methods). SIHUMI mice colonized with both *A. muciniphila* and *S.* Typhimurium had the highest macrophage infiltration scores compared to the other groups (see Figure. 1). Data are expressed as median with range. n = 5 mice per group. *P<0.05, **P<0.01, ***P<0.001. Amuc: *A. muciniphila;* S. Tm: *S.* Typhimurium.

Infection by *S.* Typhimurium involves its survival within host macrophages [23] and promotes macrophage recruitment [24], [25]. To investigate whether the presence of *A. muciniphila* enhanced this process, we scored macrophage infiltration in cecal tissue by immunohistochemical detection of the F4/80 receptor present on mouse macrophages [26]. The degree of macrophage infiltration into cecal lamina propria and submucosa was evaluated by a score ranging from 0 to 3 (as defined in the methods section). SIHUMI-AS mice displayed a significantly higher infiltration score for both lamina propria and submucosa than the SIHUMI-S, SIHUMI-A or SIHUMI mice (Figure 6). In addition, FISH analysis (Information S1) revealed that *A. muciniphila* was in close contact with the cecal epithelium in SIHUMI-A mice. In SIHUMI-S mice *S*. Typhimurium was also detected mostly on the epithelial surface whereas in SIHUMI-AS mice *S*. Typhimurium was detected deep inside the cecal tissues (Figure S4).

### Presence of *A. muciniphila* in *S.* Typhimurium-infected SIHUMI mice facilitates pathogen translocation by interfering with mucus formation

**Figure 7:**
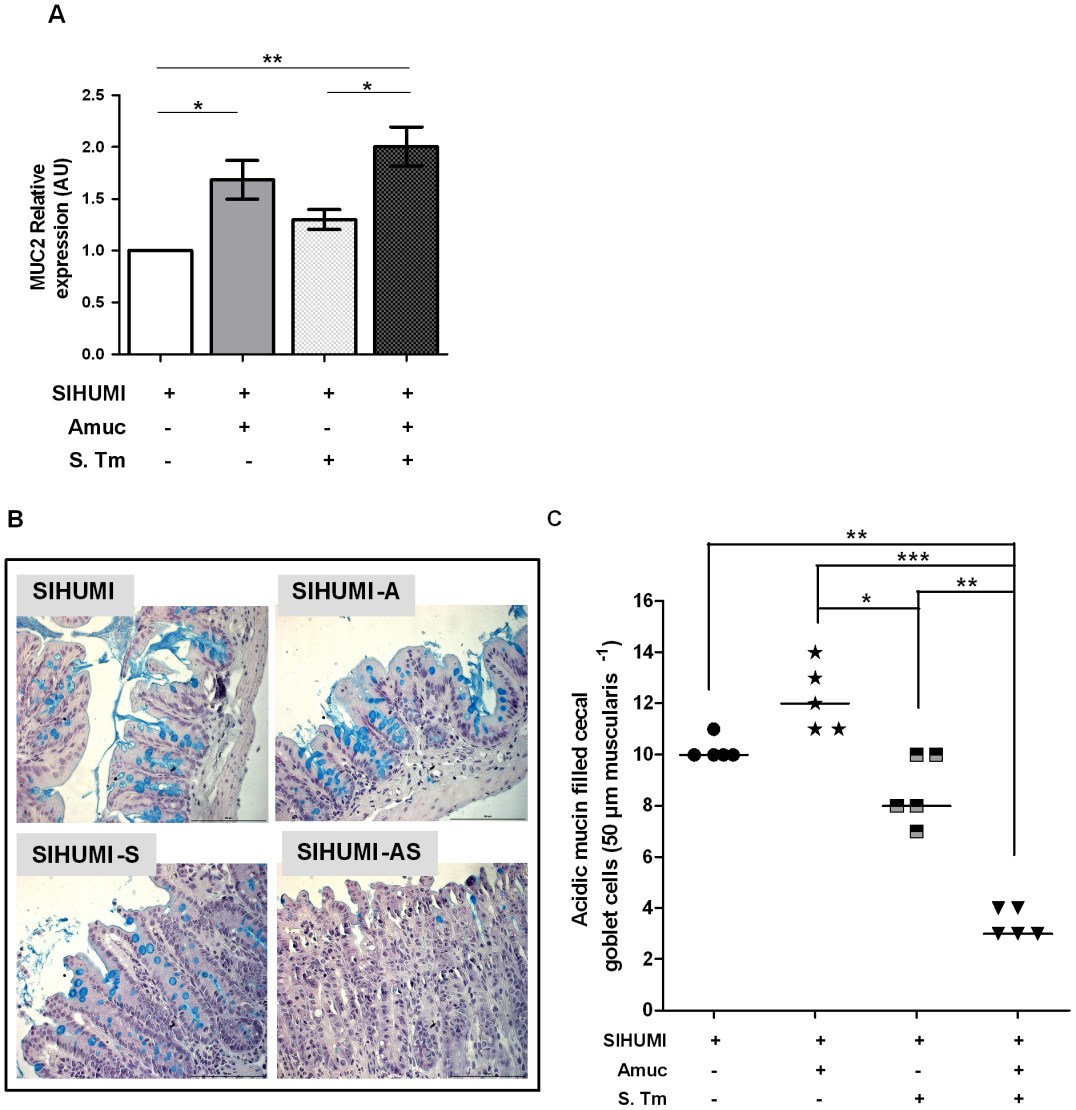
SIHUMI mice with both *A. muciniphila* and *S.* Typhimurium display increased MUC2 mRNA levels (A) and reduced numbers of mucin filled goblet cells (B and C). (A) mRNA was extracted from cecum mucosa of mice belonging to either one of four groups: SIHUMI, SIHUMI-A, SIHUMI-S and SIHUMI-AS. MUC2 mRNA from cecum mucosa was converted to cDNA and expression levels were quantified using real-time PCR (see materials and methods). SIHUMI-A and SIHUMI-AS mice showed significantly higher MUC2 gene expression compared to the other two groups, harboring no *A. muciniphila*. Data are expressed as mean±standard error. n = 6 per group. *P<0.05, **P<0.01, ***P<0.001. Amuc: *A. muciniphila;* S. Tm: *S.* Typhimurium. (B) Formalin fixed cecal tissue sections from SIHUMI, SIHUMI-A, SIHUMI-S and SIHUMI-AS mice were stained with alcian blue (pH-2.5) and haematoxylin. Images are representative of 5 mice per group. Magnification 400-fold. SIHUMI-AS mice display the lowest number of positively stained mucin-filled goblet cells compared to the other three groups. The bar represents 100 µm. (C) Quantitative analysis of the number of acidic mucin-filled goblet cells (blue) enumerated in cecal tissue sections from SIHUMI, SIHUMI-A, SIHUMI-S and SIHUMI-AS mice for a 50 µm stretch of lamina muscularis corresponding to approximately 30 cecal crypts per section. Two sections per mouse were analyzed. The number of cecal mucin filled goblet cells was elevated when *A. muciniphila* was present (SIHUMI-A) but the concomitant presence of *S.* Typhimurium (SIHUMI-AS) resulted in the lowest number of mucin filled goblet cells of gnotobiotic SIHUMI mice compared to the other mouse groups. Data are expressed as mean±standard error. n = 5 mice. *P<0.05, **P<0.01, ***P<0.001. Amuc: *A. muciniphila;* S. Tm: *S.* Typhimurium.

Since *A. muciniphila* is capable of degrading mucins, we hypothesized that this organism modified the mucus layer, which in turn enhanced exposure of the mucosa to *S.* Typhimurium, resulting in enhanced translocation of the pathogen. Stronger inflammatory and infectious symptoms in SIHUMI-AS mice compared to SIHUMI-S mice were characterized by increased cell numbers of *S.* Typhimurium in mLN, suggesting that the presence of *A. muciniphila* facilitated the translocation of the pathogen from the intestinal lumen into host tissue. We therefore investigated how the presence of *A. muciniphila* affected mucin formation, mucus thickness, mucus composition and number of mucin-filled goblet cells. Therefore, mRNA expression levels of cecal MUC2 were determined and cecum tissue sections were stained with alcian blue (AB) for quantification of goblet cells filled with acidic mucin. MUC2 gene expression was twofold higher in mice associated with *A. muciniphila* (SIHUMI-AS and SIHUMI-A mice) compared to SIHUMI-S mice or SIHUMI mice. MUC2 gene expression in the latter two groups was not significantly different (Figure 7A).

**Figure 8:**
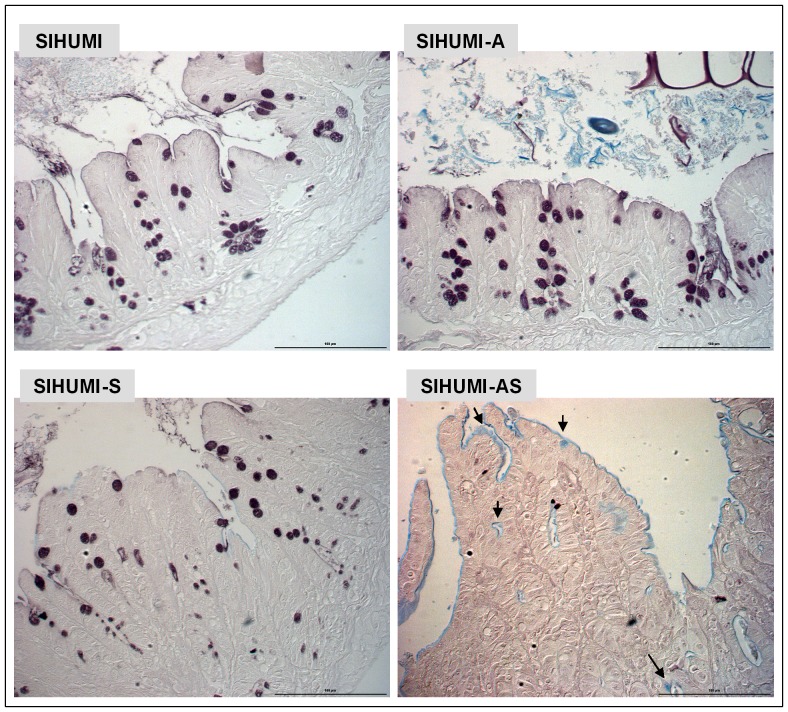
SIHUMI mice colonized with both *A. muciniphila* and *S.* Typhimurium display reduced mucus sulphation. Formalin fixed thin sections (4 µm) of cecal tissue of mice belonging to either one of four groups: SIHUMI, SIHUMI-A, SIHUMI-S and SIHUMI-AS (see Figure. 1) were stained with high iron diamine (HID)/AB at pH-2.5 and subsequently analyzed. Brown color indicates sulphated mucins while blue color indicates sialylated mucins. SIHUMI-AS mice display few sulphated mucins compared to the other mouse groups. Magnification 400×. Bars indicate 100 µm.

Higher MUC2 expression in the SIHUMI-AS mice suggested that these mice produced more mucin than the mice of the other groups. This was evaluated by staining of cecum tissue sections with AB. Microscopic examination of thin sections from cecum tissue collected 5 days p.i. revealed striking differences in the number of acidic mucin-containing goblet cells between the groups. In spite of showing the highest MUC2 expression, SIHUMI-AS mice displayed significantly lower numbers of mucin-filled goblet cells than SIHUMI-S mice or SIHUMI mice. Moreover, the cecal mucosa from SIHUMI-A mice showed the highest number of mucin-filled goblet cells compared to the mice from the other three groups (Figure 7B, 7C). Essentially the same results were obtained for colonic tissue where we observed the highest number of mucin-filled goblet cells in the SIHUMI-A group and lowest in the SIHUMI-AS group compared to the other groups (Figure S3). The cytokine patterns observed in cecum (Figure 4A) and colon (Figure S2) mucosa were very similar. Since only colonic tissue had been fixed with Carnoy's we used colonic tissue sections for investigating the impact of *A. muciniphila* on mucus layer thickness (Information S1): SIHUMI-A mice (22.3 µm±3.8 µm) had the thickest mucus layer compared to SIHUMI (9.8 µm±1.1 µm), SIHUMI-S (10.7 µm±0.8 µm) and SIHUMI-AS mice (7.5 µm±0.9 µm), whereas SIHUMI-AS mice showed 2-fold reduced mucus thickness compared to SIHUMI-A mice (Figure S3). In addition, thin sections from cecum tissue stained with high iron diamine (HID)/AB revealed a reduction in sulphated mucins in SIHUMI-AS mice compared to the mice from the other mouse groups (Figure 8).

## Discussion

### A commensal intestinal bacterium may turn into a pathobiont and contribute to an aggravation of disease symptoms

The majority of bacteria in the gastrointestinal tract are considered commensals, i.e. they do not harm the host. Our data show that the commensal *A. muciniphila* exacerbates *S.* Typhimurium-induced intestinal inflammation. However, this detrimental effect on the host can only arise under certain circumstances, in this case in the presence of both a commensal mucin degrader and a pathogen. In our study, *S.* Typhimurium-triggered inflammation turned *A. muciniphila* into a pathobiont (a resident intestinal bacterium that under certain circumstances causes disease) [27], [28]. The experiments presented here indicate that the concomitant presence of these two organisms in SIHUMI mice disturbs mucus layer homeostasis, which in turn aggravates infectious and inflammatory symptoms. The molecular interactions between a mucin-degrading commensal bacterium and a pathogenic bacterium on host health have not yet been studied and are not well understood.

The current study was triggered by previous observations in conventional IL-10^−/−^ mice treated with a probiotic *E. faecium* strain. In these mice, a significant increase in pro-inflammatory cytokine expression levels was associated with an increase in cell numbers of *A. muciniphila* [13]. The results presented herein are in accordance with these observations because the latter organism also affected inflammatory parameters in our present study. For example, mRNA expression levels of IFN-γ, IP-10, TNF-α, IL-6, IL-12 and IL-17 were increased in SIHUMI-AS compared to SIHUMI-S mice. It may be concluded that *S.* Typhimurium alone leads to a considerably weaker gut inflammation as compared to when *A. muciniphila* is also present.

IL-12 and IL-18 have been described to increase in response to a *S.* Typhimurium infection and in turn to induce the production of IFN-γ which enhances the ability of macrophages to kill intracellular pathogens [25], [29]. Interestingly, in our experiment, we only observed an up-regulation of IL-12 in the SIHUMI-AS mice, while IL-18 was significantly down-regulated in these mice compared to the other groups. IL-12 formation by infected macrophages is an important defense against *Salmonella* because it leads to the recruitment of Natural Killer (NK) cells to the infected site, a higher production of IFN-γ, and in turn an enhanced differentiation of monocytes to macrophages [30]–[32]. In line with these studies, we observed significantly higher numbers of cecal macrophages accompanied by higher cecal and colonic mRNA levels of IL-12 and IFN-γ in SIHUMI-AS compared to SIHUMI-S mice. *S.* Typhimurium survives and grows inside macrophages from where the pathogen invades host tissues [33]. In accordance with the elevated IL-12 and IFN-γ mRNA levels *S.* Typhimurium cell numbers were 10 fold higher in mLN of SIHUMI-AS compared to SIHUMI-S mice. IL-18 in conjunction with IL-12 is involved in phagocytosis of intracellular pathogens [29]. The observed suppression of IL-18 in SIHUMI-AS compared to SIHUMI-S mice, led us to speculate that the concomitant presence of *A. muciniphila* and *S.* Typhimurium facilitates growth of the pathogen in the infected macrophages because down-regulation of IL-18 protects *S.* Typhimurium from being killed. In addition, SIHUMI-AS mice showed significantly higher cecal histopathology scores compared to SIHUMI-S infected mice. Contrary to cecum, the colon displayed 5 days p.i. no elevated histopathology scores in SIHUMI-AS mice and SIHUMI-S mice. This may be explained by the fact that it takes several days for the inflammation to spread from cecum to colon [34].

### *A. muciniphila*'s ability to disturb host mucus-homeostasis appears crucial for its ability to exacerbate infectious and inflammatory symptoms caused by *S.* Typhimurium

Commensal bacterium *A. muciniphila* is known for its ability to degrade mucins [16], [17]. Recent studies propose that excessive mucin degradation facilitates the access of pathogen to the mucosa [12], [35]. The experiments presented in this paper support the view that the presence of the mucin-degrading *A. muciniphila* causes an aggravation of intestinal inflammatory symptoms caused by *S.* Typhimurium infection. Using a consortium of eight bacterial species [18] as a background microbiota we observed that the concomitant presence of *A. muciniphila* and *S.* Typhimurium resulted in mucus-related differences that were absent or less pronounced if either one of the strains was present. For example, the number of mucin-filled goblet cells in SIHUMI-AS mice was 2.5- to 4- fold lower than in any other of the mouse groups. Paradoxically, the MUC2 gene expression level in the SIHUMI-AS mice was higher than that in the SIHUMI-S or the SIHUMI mice. Why higher mRNA levels of MUC2 in mucosal tissue did not coincide with higher numbers of mucin-filled goblet cells is not quite clear. Two explanations are conceivable: 1. Previous studies indicate that severe inflammation causes endoplasmic reticulum (ER) stress in intestinal epithelial cells and in goblet cells [36]–[40]. For example, a ribotoxic stress response caused apoptosis of intestinal epithelial cells triggered by Shiga toxin-producing *E. coli* [41] and of goblet cells [42], [43]. Such stress acting on goblet cells might result in increased expression of the MUC2 gene to compensate for the loss of mucin-filled goblet cells. However, owing to cellular stress, decoration of the mucin polypeptide backbone with carbohydrates would remain fragmentary. Since AB does not stain the mucin polypeptide backbone, undecorated mucin would therefore not be detectable with AB in goblet cells. 2. Previous findings demonstrated that infection with *S.* Typhimurium enhances mucin excretion from goblet cells by increased expression of IFN-γ [44]. Therefore, the 2.5- fold higher IFN-γ expression level in SIHUMI-AS mice relative to SIHUMI-S mice may have led to an emptying of goblet cells to restrict the load of pathogens in the host. In support of this assumption we observed a significant inverse correlation (r^2^ =  −0.86, *P<0.001*) between the number of mucin filled goblet cells and IFN-γ gene expression levels (data not shown).

Mucus is constantly secreted into the intestine, where it forms a protective gel-like structure of approximately 150 µm thicknesses on the mucosal surface. Cecum and colon mucosa is covered with a tightly packed inner mucus layer and a less dense outer layer. The inner layer serves as a barrier that prevents bacterial access to the epithelium [45]. Even though the inner mucus layer is usually devoid of bacteria, we detected *A. muciniphila* in close contact with the cecal epithelial surface in the SIHUMI-A mice. We speculate that *A. muciniphila* promotes mucin formation and thereby supports its own growth via mucin degradation similar to what has been observed for *B*. *thetaiotaomicron* in NMRI mice; utilization of fucose by this organism triggered the synthesis of fucosylated glycoconjugates by the host epithelium [5].

The reduced brown color observed after HID/AB (pH-2.5) staining in cecal tissue sections of SIHUMI-AS mice compared to those of the other mouse groups indicate a loss of mucin sulphation in this group. Interestingly, changes in intestinal mucin composition characterized by a lower degree of sulphation [7], [46] and a higher degree of sialylation have previously been reported to occur in UC, Crohn's Disease (CD) and gastric ulcer caused by *Helicobacter pylori*-induced inflammation [7], [46], [47]. These changes might facilitate access of intraluminal antigens and thereby possibly aggravate inflammatory symptoms.

### Presence of both *A. muciniphila* and *S.* Typhimurium is associated with drastic changes in microbiota composition

The above findings indicate that the presence of *A. muciniphila* within the SIHUMI-AS consortium is responsible for the exacerbation of inflammation observed in the corresponding mice. One of the most prominent differences between *S.* Typhimurium-infected SIHUMI mice with or without *A. muciniphila* relates to drastic differences in microbiota composition. The data indicate that *A. muciniphila* promotes the growth of *S.* Typhimurium, which reaches a proportion of 94% in the presence of *A. muciniphila* compared to 2.2% in its absence, while other community members including *B. thetaiotaomicron* and *E. coli* decreased dramatically from 88% to 0.02% and 0.14% to 0.01%, respectively. The reasons for this dramatic change are not really known. It may be speculated that this phenomenon is related to *A. muciniphila*'s ability to exacerbate *S.* Typhimurium-induced inflammation whereas the presence of *A. muciniphila* is without consequence when *S.* Typhimurium is absent. Presence of either *A. muciniphila* or *S.* Typhimurium alone did not lead to such a dramatic shift in the existing microbiota composition as evident from a comparison of SIHUMI, SIHUMI-A and SIHUMI-S mice, which showed no major differences in the relative proportions of the SIHUMI community members between these mouse groups. Enteropathogenic bacteria such as *S.* Typhimurium are known to breach colonization resistance and to invade host tissues by exploiting host inflammation [14], [48], [49]. Higher numbers of *S.* Typhimurium in mesenteric lymph nodes of the SIHUMI-AS mice compared to SIHUMI-S mice suggest that *A. muciniphila* contributes to an impairment of colonization resistance and enhances intestinal inflammation. In fact, we observed higher mRNA levels of pro-inflammatory markers in SIHUMI-AS compared to SIHUMI-S mice.

We propose that the enhanced inflammatory host response in the SIHUMI-AS mice was responsible for the dramatic decrease in the *B. thetaiotaomicron* population. We speculate that the decimation of *B. thetaiotaomicron* might be due to the generation of higher concentrations of reactive oxygen and nitrogen species [25], [50] in the more severely inflamed SIHUMI-AS mice compared to SIHUMI-S mice.

The reduction of *E. coli* numbers in the SIHUMI-AS versus the SIHUMI-S mice was moderate compared to that of *B. thetaiotaomicron* and is in contradiction to previous studies where an increase in *E. coli* was observed in conventional mice in response to a *S.* Typhimurium-induced gut inflammation [14], [50] or in inflamed IL-10^−/−^ mice [51]. One possible explanation for reduced *E. coli* cell numbers in the SIHUMI-AS mice could be due to the fact that we used a non-pathogenic laboratory strain of *E. coli* which lacks fitness genes [52] and might therefore be more susceptible to inflammatory conditions.

Composition of the microbiota of IBD patients significantly differs from that in healthy controls [53]. Currently, an imbalance in gut microbiota is regarded as one possible factor triggering the inflammation in UC and CD [3], [6], [10]. Our data suggest that the presence of a dedicated mucin-degrading bacterium supports a pathogen-induced inflammation, which in turn leads to alterations in the existing gut microbiota composition.

A possible limitation of our mouse model lies in the use of a simplified human intestinal microbiota, which does not completely reflect the features of a conventional microbiota. Therefore, we cannot directly extrapolate the observed effects from the SIHUMI-AS mice to conventional mice. However, in spite of these limitations this model offers the chance to identify the molecular mechanisms underlying the interactions between a pathogen, a commensal microbiota and the host because each SIHUMI member is known and can be tracked.

**Figure 9:**
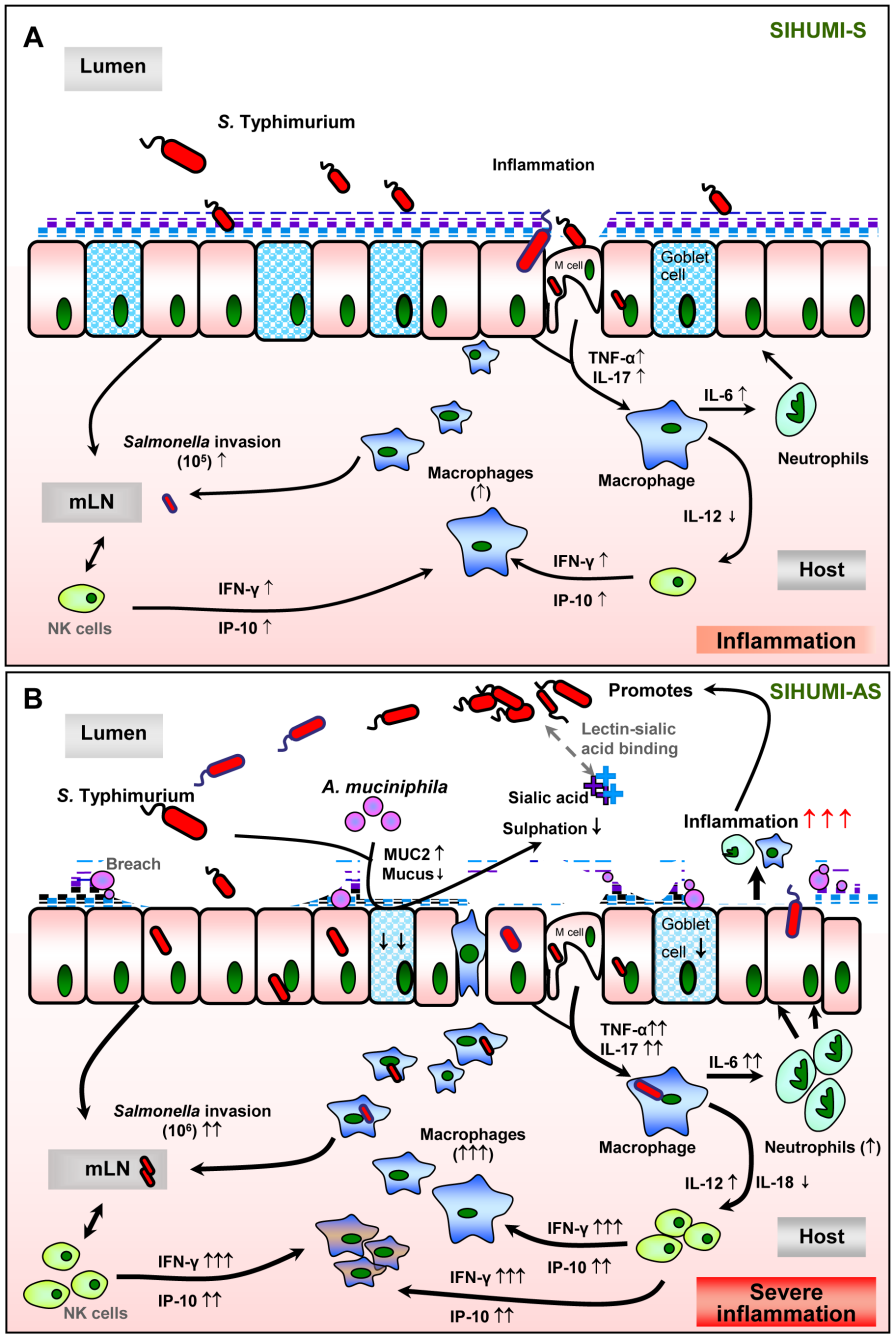
Hypothetical Scheme. The presence of *A. muciniphila,* leads to the exacerbation of *S.* Typhimurium-induced intestinal inflammation. We propose that the presence of *A. muciniphila* causes changes in mucin composition and production, which in turn facilitates the invasion of *S.* Typhimurium into the host. Increased inflammatory status was characterized by increased pro-inflammatory cytokines, increased macrophage infiltration and invasion of the pathogen into the lymph nodes, reduced number of mucin-filled goblet cells in SIHUMI-AS mice (B) compared to SIHUMI-S mice (A). Our data suggests that in the presence of both *A. muciniphila* and *S.* Typhimurium, mucus sulphation is diminished and this may facilitate the access of *S.* Typhimurium to sialic acid in mucus. Sialic acid may serve as a substrate and adhesion site for *S.* Typhimurium in the gut [56], [57]. Increased gene expression of IFN-γ and IP-10 indicate an increased NK-cell recruitment. mLN - mesenteric lymph nodes, NK- Natural killer cells. (↑ increased; ↓ decreased; grey dotted line: assumed processes including lectin-sialic acid binding [56], M-cells for pathogen transit [43], [58], [59]; black line: supported by data of the present study).

Taken together our experiments indicate that *A. muciniphila* facilitates infection by *S.* Typhimurium in mice colonized with a simplified human intestinal microbiota and thereby exacerbates infectious and inflammatory symptoms. This was not the case in SIHUMI mice colonized with *S.* Typhimurium in the absence of *A. muciniphila* (Figure 9). This is an impressive example on how a community member changes its role in the ecosystem in response to the presence of a pathogen and how it shifts from a commensal to a harmful bacterium (pathobiont).

## Materials and Methods

### Bacterial Strains

The bacterial strains used in this study were: *A. muciniphila* ATCC BAA-835 and *S.* Typhimurium DT104 as well as members of a simplified human intestinal microbiota (SIHUMI) consisting of eight bacterial species *(Bifidobacterium longum* NCC 2705, *Blautia producta* DSM 2950, *Bacteroides thetaiotaomicron* DSM 2079, *Clostridium ramosum* DSM 1402, *Clostridium butyricum* DSM 10702, *Escherichia coli* K-12 MG1655, *Lactobacillus plantarum* DSM 20174 and *Anaerostipes caccae* DSM 14662*)*. All strains were routinely cultured at 37°C. The SIHUMI members and *S.* Typhimurium were cultured in yeast casitone fatty acid (YCFA) medium [18] while *A. muciniphila* was cultured in Columbia broth (Difco). All strains were cultured under strictly anoxic conditions using N_2:_CO_2_ (80∶20; v∶v) as the gas phase.

### Animal experiment

Germ-free C3H mice were bred in Trexler-type isolators. After weaning, all mice were colonized by gavaging the fecal supernatant of SIHUMI mice [18]. Forty of these SIHUMI mice were allocated to four groups (10 mice per group). The groups differed in their microbial status: they were subsequently colonized with *A. muciniphila* (SIHUMI-A) or with *S.* Typhimurium (SIHUMI-S) or with both *A. muciniphila* and *S.* Typhimurium (SIHUMI-AS) as indicated in Figure 1. The bacteria were grown anaerobically overnight at 37°C and their cell numbers were determined using a counting chamber. Mice were inoculated with: 5*10^7^ cells of *S.* Typhimurium suspended in 200 µl medium and 1*10^8^ cells of *A. muciniphila* suspended in 200 µl medium by gastric gavage. Mice colonized with only SIHUMI members received 200 µl of sterile medium. Successful bacterial colonization after inoculation was validated in the feces by qPCR (see below).

The animals were killed by cervical dislocation at the times indicated in Figure 1. Cecal and colonic contents were collected and bacterial cells were enumerated by qPCR. Spleen and mesenteric lymph nodes were collected for enumeration of *S.* Typhimurium. Colon tissue samples were fixed in formalin and Carnoy's solution for histochemical analysis and measurement of mucus thickness. Cecum tissue samples were fixed with formalin and embedded in paraffin (p) for immunohistochemical analysis (IHC-p), stained with haematoxylin and eosin (H&E) for histopathology scoring and with alcian blue (AB) for enumeration of mucin-filled goblet cells. Bacterial cells were detected by 16S rRNA-targeted fluorescence in-situ hybridization (FISH). Five of the 10 mice per group were used for colon mucosa scrapings while colon tissue of the remaining 5 mice was subjected to Carnoy's fixation. In addition, cecal tissue from all 10 mice per group was used in part for mucosa scrapings and in part subjected to formalin fixation. Scraped mucosa samples were flash frozen in liquid nitrogen and stored at −80°C until use. Approximately 25 mg of the frozen intestinal mucosa scrapings were homogenized for RNA extraction. Blood was collected for measuring serum inflammatory biomarkers using ELISA. All samples were frozen at −80°C until use.

### Ethics statement

The protocol for the animal experiment was approved by the Animal Welfare Committee of the Ministry of Environment, Health and Consumer Protection of the Federal State of Brandenburg (Germany), State Office of Environment, Health and Consumer Protection (approval number: V3-2347-42-2011). The regulations of the German Animal Welfare Act (TierSchG, §8, Abs.1) were strictly followed.

### Quantification of bacterial cell numbers

Bacterial DNA was extracted from cecal, colonic and fecal sample material using the PSP Spin Stool DNA plus Kit (Invitek, Berlin, Germany) and used for the quantification of *A. muciniphila*, *S.* Typhimurium and the members of the SIHUMI consortium present in the gut contents. Bacteria were quantified using quantitative Real-Time PCR targeting the 16S rRNA gene in the case of *A. muciniphila* [13], the ttr (tetrathionate respiration) - region in the case of *S.* Typhimurium, as described previously [54] and the HSP-60 gene for each member of the SIHUMI consortium [55]. All primers were purchased from MWG Eurofins (Germany). Standard curves were obtained by spiking feces from germfree mice with known cell numbers of *A. muciniphila*, *S.* Typhimurium or individual SIHUMI bacteria. The Applied Biosystems 7500 FAST Real-Time PCR (Life Technologies GmbH, Darmstadt, Germany) was used for amplification and fluorescent data collection. The supplied software was used to calculate absolute cell numbers according to the calibration curves. The master mix consisted of 12.5 µl QuantiFast SYBR Green 2000 (Qiagen, Hilden, Germany), 0.5 µl of each primer (10 µM), 1 µl of sample and adjusted with water to a final volume of 25 µl per well. After PCR amplification, the specificity of the primers was checked by inspecting the melting curve and determining the size of the amplicon by agarose gel electrophoresis (1%). Bacterial DNA from mesenteric lymph nodes (mLN) and spleen was extracted with the Blood and Tissue DNA extraction kit (Qiagen) and used for the quantification of *S.* Typhimurium. Standards were obtained by spiking homogenized mLN or spleen of SIHUMI mice with known cell numbers of *S.* Typhimurium.

### Evaluation of intestinal inflammation

Formalin fixed cecal and colonic sample material was embedded in paraffin and sectioned at 4 µm. After staining with haematoxylin and eosin, gut inflammation was evaluated by an experienced pathologist in a blinded fashion. The histopathology scores was based on the following parameters: villous atrophy and fusion: 1 =  scant (ca. 10); 2 =  moderate; 3 =  dense, lymphocytes: 0 =  one small; 1 =  some (2–4); 3 =  numerous (>5) or 1 large, necrotic epithelial cells: 1 =  scant (ca. 10); 2 =  moderate; 3 =  dense, PMN: 0 =  none; 1 =  few extravascular PMNs; 2 =  many polymorph nuclear cells (neutrophils), neutrophils: 1 =  Scant (1); 2 =  moderate, 3 =  dense, infiltration: 0 =  none; 1 = rare (<15%); 2 =  moderate; 3 =  abundant (>50%), desquamation: 1 =  Patchy (<30%); 2 =  diffuse (> 30%), edema: 0 =  none to mild (<10 of the mucosa); 1 =  moderate; 2 =  severe, ulceration: 0 =  no; 1 =  present, Crypt abscesses: 0 =  none; 1 =  rare; 2 =  moderate; 3 =  abundant, Peyer patch hyperplasia: 0 =  none; 1 =  present and epithelial hyperplasia: 0 =  none; 1: present.

### mRNA levels of pro-inflammatory cytokines from intestinal mucosa samples

To quantify the relative mRNA expression levels of interferon (IFN)-γ, tumor necrosis factor (TNF)-α, interferon gamma-inducible protein (IP)-10, interleukin (IL)-6, IL-12, IL-23, IL-17, IL-18 and IL-4, RNA was extracted from intestinal mucosa samples using the miRNeasy mini kit (Qiagen, Hilden, Germany). One µg of RNA was reverse-transcribed to single-stranded cDNA using the RevertAid H minus First Strand cDNA Synthesis Kit (Fermentas, St. Leon-Roth, Germany). Reverse transcriptase real-time (RT) PCR was performed using the Applied Biosystems 7500 Fast Real-Time PCR system (Life Technologies GmbH). The RT-PCR reaction mix (adjusted with H2O to a total volume of 25 µl) contained 1 µl template DNA, 12.5 µl QuantiFast SYBR Green PCR master mix (Qiagen), 0.5 µl of the respective primers (10 µM each). The forward and reverse primers used for IFN-γ, IP-10, IL-12, IL-17, TNF-α, IL-6, IL-23, IL-18 and IL-4 quantification were described previously [13]. MUC2 forward (5'-GTGGCTGCGTGCCTAGTCCT-3') and reverse primers (5'-AGGCCGGCCCGAGAGTAGAC-3') were designed using Primer BLAST (NCBI). Relative mRNA target gene expression levels (Ratio = [(E_target_) ^dCPtarget (control-sample)^]/[(E_ref._) ^dCPref. (control-sample)^]) were normalized to the house keeping gene glyceraldehyde 3-phosphate dehydrogenase (GAPDH) and used as a reference. Subsequently, intestinal mucosal cytokine and MUC2 gene expression values of the SIHUMI group were set to 1.0 and used as the calibrator to identify the relative mRNA fold difference between the SIHUMI, SIHUMI-A, SIHUMI-S and SIHUMI-AS groups.

### Measurement of cytokines in blood plasma using ELISA

Serum levels of IFN-γ, TNF-α and IL-6 concentrations were measured in duplicate by enzyme-linked immunosorbent assay (ELISA) using a commercially available kit (Abcam, Cambridge, UK). The concentrations were calculated from standard curves according to the manufacturer's instruction. The detection limits for the aforementioned cytokines were 46.9 pg/ml, 31.3 pg/ml and 8.06 pg/ml, respectively.

### Immunohistochemical analysis

Formalin-fixed, paraffin-embedded cecal sections (2 µm) were incubated overnight at 4°C with a primary antibody targeting the mouse macrophage-specific receptor F4/80 (Abcam, Cambridge, UK) after antigen retrieval according to the manufacturer's instructions. Samples were washed and subsequently incubated with Histofine (anti-rat secondary antibody fab‘ fragment from Nichirei, Tokyo, Japan) for 30 min at RT. Immunoperoxidase staining was performed with the diaminobenzidine substrate kit (Sigma-Aldrich, Munich, Germany). Sections were counterstained with haematoxylin and examined by light microscopy in a blinded fashion. Approximately, 50 µm of cecal lamina muscularis corresponding to approximately 30 crypts per mouse and per section were scored. The scores represent positively stained cells in lamina propria and sub-mucosa as follows, 0 =  none (between 0 and 4), 1 =  normal (between 5 and 8), 2 =  moderate (between 9 and 12) and 3 =  severe (between 13 and above). The scores are shown individually for lamina propria and sub-mucosa.

### Alcian blue staining of cecal tissue samples

The formalin-fixed cecal tissue was sectioned at 4 µm and stained with alcian blue (AB) at pH-2.5, which stains acidic mucins blue. Goblet cells were enumerated in a 50 µm stretch of the lamina muscularis corresponding to approximately 30 crypts per section and per mouse using an Eclipse E600 microscope (NIKON, Germany) and inspecting the images captured with a MV-1500 digital camera and Lucia G software version 4.51 (Laboratory imaging s.r.o.) for Windows 7 (Microsoft, Munich, Germany) was used. To distinguish different mucins, colonic and cecal tissue sections were stained with periodic acid Schiff (PAS)/AB. Cecal tissue sections were additionally stained with high iron diamine (HID)/AB at pH-2.5, which stains sulphated mucins (sulphomucin) brown and sialylated mucins (sialomucin) blue. Images were analyzed using an Eclipse E600 microscope and captured with a MV-1500 digital camera (NIKON, Düsseldorf, Germany).

### Statistical analysis

Data were tested for normal distribution using the Kolmogorov–Smirnov test. Normally distributed data are presented as means with standard error while the medians with their range are given for non-normally distributed data. Significance of differences between SIHUMI, SIHUMI-S, SIHUMI-A and SIHUMI-AS mice were analyzed using the One-way analysis of variance test for normally distributed data (or) the Kruskal-Wallis test for non-normally distributed data, followed by either Bonferroni/Tukey or Dunn's comparison post-hoc tests. Differences between SIHUMI-S and SIHUMI-AS mice were analyzed using students t-test followed by the Mann-Whitney test for non-normally distributed data. The correlation between IFN-γ expression levels and number of mucin filled goblet cells in the mice were analyzed by the Pearson correlation coefficient test. Differences between the groups were considered significant at *P < 0·05, **P<0.01, ***p<0.001. SPSS 16.0 (IBM, Munich, Germany) for Windows 7 was used for data analysis. Prism 5.0 software (Graph Pad Software, Inc., La Jolla, CA, USA) for Windows, was used for data presentation.

## Supporting Information

Figure S1 **Presence of** ***A. muciniphila*** **renders** ***S.*** **Typhimurium the dominant species in colon of gnotobiotic SIHUMI mice.** Colonic contents were recovered from gnotobiotic C3H mice assigned to 4 groups, differing in their microbial status: (A) Mice with a defined microbial community of eight bacterial species (SIHUMI), (B) SIHUMI mice colonized additionally with *A. muciniphila* (SIHUMI-A), (C) SIHUMI mice infected with *S.* (SIHUMI-S) and (D) SIHUMI mice colonized with *A. muciniphila* and 10 days later infected with *S.* Typhimurium (SIHUMI-AS). Total DNA was extracted and bacterial cell numbers were quantified by primers targeting the HSP60 gene of the SIHUMI members, the 16S rRNA gene of *A. muciniphila* and the ttr-region of *S.* Typhimurium using quantitative PCR. Calculation of the cell numbers was based on DNA obtained from cell suspensions containing known cell numbers of the targeted bacterial species (see materials and methods). Reduced proportion of SIHUMI members in SIHUMI-AS mice is attributed to an increase in the proportion of *S.* Typhimurium cells. Ten animals per group were used. The exact bacterial cell numbers and *P-values* for the differences between the groups are provided in Table S1.
(TIF)

Figure S2 **Presence of both** ***A. muciniphila*** **and** ***S.*** **Typhimurium is accompanied by increased colonic pro-inflammatory cytokine mRNA levels.** Colonic mRNA levels of IFN-γ, IP-10, TNF-α, IL-12, IL-6 and IL-17 in gnotobiotic C3H mice were measured. mRNA was extracted from colon mucosa of mice belonging to either one of four groups: SIHUMI, SIHUMI-A, SIHUMI-S and SIHUMI-AS (see Figure. 1). The mRNA was converted to cDNA for quantitative real-time PCR measurement (see materials and methods). Inoculation of the gnotobiotic SIHUMI mice with *A. muciniphila* followed by *S.* Typhimurium infection (SIHUMI-AS) caused an increase in mRNA levels of pro-inflammatory cytokines. Data are expressed as mean±standard error. n = 6 per group. Star indicates statistically significant differences (*P<0.05, **P<0.01, ***P<0.001). AU: Arbitrary units. (Amuc: *A. muciniphila;* S. Tm: *S.* Typhimurium).
(TIF)

Figure S3 **Presence of both *A. muciniphila* and *S.* Typhimurium caused reduction in number of mucin filled goblet cells in colon of SIHUMI mice.** Carnoy-fixed cecal tissue sections (4 µm) from SIHUMI, SIHUMI-A, SIHUMI-S and SIHUMI-AS (see Figure. 1) mice were stained with periodic acid Schiff/Alcian blue (PAS/AB) at both pH 2.5 and pH 1.0. Images are representative of 5 mice per group. (A-i) All acidic mucins are stained blue with AB at pH 2.5 whereas all neutral mucins are stained magenta with PAS; (A-ii) highly sulphated mucins are stained blue with AB at pH-1. All the images from (A-i & A-ii) are obtained with a magnification of 1000-fold. Bars indicate 20 µm. (B-i) colonic tissues stained with PAS/AB at pH 2.5; (B-ii) colonic tissues stained with PAS/AB at pH 1.0 obtained with a magnification of 400-fold. Bars indicate 100 µm. SIHUMI-AS mice display the lowest number of positively stained colonic mucin-filled goblet cells compared to the other three groups at any given pH. L: lumen.
(TIF)

Figure S4 **Detection of** ***A. muciniphila*** **and** ***S.*** **Typhimurium attached to mucosa in cecal tissue section by FISH.** Thin sections (4 µm) of formalin fixed cecal tissue were used for the detection of *A. muciniphila* and *S.* Typhimurium by fluorescence in-situ hybridization (FISH) in gnotobiotic mice belonging to either one of the four groups: SIHUMI, SIHUMI-A, SIHUMI-S and SIHUMI-AS (see Figure. 1). Thin sections were hybridized with Cy3 labeled oligonucleotide probes (see Information S1) targeting *A. muciniphila* (S-S-MUC-1437-a-A-20) *at* 55°C and *S.* Typhimurium (L-S-Sal-1713-a-A-18) at 45°C. DNA was counterstained with 4',6-diamidino-2-phenylindole (DAPI). (A) *A. muciniphila* is in close contact to the epithelial surface in SIHUMI-A mice. (B) *S.* Typhimurium cells are found mostly on the epithelial cell surface of SIHUMI-S mice. (C) *S.* Typhimurium is in cecal tissue of SIHUMI-AS mice. Magnification 1000×. The scales represent 20 µm. L: lumen.
(TIF)

Information S1 Supporting Materials and Methods.
(PDF)

Table S1 *S.* Typhimurium becomes the dominant species in colon of SIHUMI mice previously associated with *A. muciniphila*.
(PDF)

## References

[1] Derrien M, van Passel MW, van de Bovenkamp JH, Schipper RG, de Vos WM, et al (2010) Mucin-bacterial interactions in the human oral cavity and digestive tract. Gut Microbes 1: 254–268. 21327032 10.4161/gmic.1.4.12778 PMC3023607

[2] Kim YS, Ho SB (2010) Intestinal goblet cells and mucins in health and disease: recent insights and progress. Curr Gastroenterol Rep 12: 319–330. 20703838 10.1007/s11894-010-0131-2 PMC2933006

[3] Sartor RB (2009) Microbial-host interactions in inflammatory bowel diseases and experimental colitis. Nestle Nutr Workshop Ser Pediatr Program 64: 121–132; discussion 132–127, 251–127. 10.1159/000235787 19710519

[4] Swidsinski A, Loening-Baucke V, Theissig F, Engelhardt H, Bengmark S, et al (2007) Comparative study of the intestinal mucus barrier in normal and inflamed colon. Gut 56: 343–350. 16908512 10.1136/gut.2006.098160 PMC1856798

[5] Falk PG, Hooper LV, Midtvedt T, Gordon JI (1998) Creating and maintaining the gastrointestinal ecosystem: What we know and need to know from gnotobiology. Microbiology and Molecular Biology Reviews 62: 1157-+. 10.1128/mmbr.62.4.1157-1170.1998 PMC98942 9841668

[6] Campieri M, Gionchetti P (2001) Bacteria as the cause of ulcerative colitis. Gut 48: 132–135. 11115835 10.1136/gut.48.1.132 PMC1728175

[7] Corfield AP, Myerscough N, Longman R, Sylvester P, Arul S, et al (2000) Mucins and mucosal protection in the gastrointestinal tract: new prospects for mucins in the pathology of gastrointestinal disease. Gut 47: 589–594. 10986224 10.1136/gut.47.4.589 PMC1728059

[8] Duerkop BA, Vaishnava S, Hooper LV (2009) Immune Responses to the Microbiota at the Intestinal Mucosal Surface. Immunity 31: 368–376. 19766080 10.1016/j.immuni.2009.08.009

[9] Gassler N, Rohr C, Schneider A, Kartenbeck J, Bach A, et al (2001) Inflammatory bowel disease is associated with changes of enterocytic junctions. American Journal of Physiology-Gastrointestinal and Liver Physiology 281: G216–G228. 11408275 10.1152/ajpgi.2001.281.1.G216

[10] Sartor RB (2006) Mechanisms of disease: pathogenesis of Crohn's disease and ulcerative colitis. Nat Clin Pract Gastroenterol Hepatol 3: 390–407. 16819502 10.1038/ncpgasthep0528

[11] Sekirov I, Russell SL, Antunes LC, Finlay BB (2010) Gut microbiota in health and disease. Physiol Rev 90: 859–904. 20664075 10.1152/physrev.00045.2009

[12] Wiggins R, Hicks SJ, Soothill PW, Millar MR, Corfield AP (2001) Mucinases and sialidases: their role in the pathogenesis of sexually transmitted infections in the female genital tract. Sexually Transmitted Infections 77: 402–408. 11714935 10.1136/sti.77.6.402 PMC1744407

[13] Ganesh BP, Richter JF, Blaut M, Loh G (2012) Enterococcus faecium NCIMB 10415 does not protect interleukin-10 knock-out mice from chronic gut inflammation. Beneficial Microbes 3: 43–50. 22348908 10.3920/BM2011.0050

[14] Stecher B, Robbiani R, Walker AW, Westendorf AM, Barthel M, et al (2007) Salmonella enterica serovar typhimurium exploits inflammation to compete with the intestinal microbiota. PLoS Biol 5: 2177–2189. 17760501 10.1371/journal.pbio.0050244 PMC1951780

[15] Collado MC, Derrien M, Isolauri E, de Vos WM, Salminen S (2007) Intestinal integrity and Akkermansia muciniphila, a mucin-degrading member of the intestinal microbiota present in infants, adults, and the elderly. Appl Environ Microbiol 73: 7767–7770. 17933936 10.1128/AEM.01477-07 PMC2168041

[16] Derrien M, Collado MC, Ben-Amor K, Salminen S, de Vos WM (2008) The mucin degrader Akkermansia muciniphila is an abundant resident of the human intestinal tract. Applied and Environmental Microbiology 74: 1646–1648. 18083887 10.1128/AEM.01226-07 PMC2258631

[17] Derrien M, Vaughan EE, Plugge CM, de Vos WM (2004) Akkermansia muciniphila gen. nov., sp. nov., a human intestinal mucin-degrading bacterium. Int J Syst Evol Microbiol 54: 1469–1476. 15388697 10.1099/ijs.0.02873-0

[18] Becker N, Kunath J, Loh G, Blaut M (2011) Human intestinal microbiota: characterization of a simplified and stable gnotobiotic rat model. Gut Microbes 2: 25–33. 21637015 10.4161/gmic.2.1.14651

[19] Zirk NM, Hashmi SF, Ziegler HK (1999) The polysaccharide portion of lipopolysaccharide regulates antigen-specific T-cell activation via effects on macrophage-mediated antigen processing. Infect Immun 67: 319–326. 9864232 10.1128/iai.67.1.319-326.1999 PMC96313

[20] Bruno VM, Hannemann S, Lara-Tejero M, Flavell RA, Kleinstein SH, et al (2009) Salmonella Typhimurium type III secretion effectors stimulate innate immune responses in cultured epithelial cells. PLoS Pathog 5: e1000538. 19662166 10.1371/journal.ppat.1000538 PMC2714975

[21] Mathur R, Oh H, Zhang D, Park SG, Seo J, et al (2012) A mouse model of Salmonella typhi infection. Cell 151: 590–602. 23101627 10.1016/j.cell.2012.08.042 PMC3500584

[22] Winter SE, Thiennimitr P, Winter MG, Butler BP, Huseby DL, et al (2010) Gut inflammation provides a respiratory electron acceptor for Salmonella. Nature 467: 426–429. 20864996 10.1038/nature09415 PMC2946174

[23] Monack DM, Bouley DM, Falkow S (2004) Salmonella typhimurium persists within macrophages in the mesenteric lymph nodes of chronically infected Nramp1(+/+) mice and can be reactivated by IFN gamma neutralization. Journal of Experimental Medicine 199: 231–241. 14734525 10.1084/jem.20031319 PMC2211772

[24] Nix RN, Altschuler SE, Henson PM, Detweiler CS (2007) Hemophagocytic macrophages harbor Salmonella enterica during persistent infection. Plos Pathogens 3: 1982–1992. 10.1371/journal.ppat.0030193 PMC2134957 18085823

[25] Thiennimitr P, Winter SE, Baumler AJ (2012) Salmonella, the host and its microbiota. Current Opinion in Microbiology 15: 108–114. 22030447 10.1016/j.mib.2011.10.002 PMC3265626

[26] Kallis YN, Robson AJ, Fallowfield JA, Thomas HC, Alison MR, et al (2011) Remodelling of extracellular matrix is a requirement for the hepatic progenitor cell response. Gut 60: 525–533. 21106552 10.1136/gut.2010.224436

[27] Ayres JS, Trinidad NJ, Vance RE (2012) Lethal inflammasome activation by a multidrug- resistant pathobiont upon antibiotic disruption of the microbiota. Nature Medicine 18: 799–U201. 10.1038/nm.2729 PMC3472005 22522562

[28] Chow J, Mazmanian SK (2010) A Pathobiont of the Microbiota Balances Host Colonization and Intestinal Inflammation. Cell Host & Microbe 7: 265–276. 20413095 10.1016/j.chom.2010.03.004 PMC2859213

[29] Berclaz PY, Shibata Y, Whitsett JA, Trapnell BC (2002) GM-CSF, via PU.1, regulates alveolar macrophage Fcgamma R-mediated phagocytosis and the IL-18/IFN-gamma -mediated molecular connection between innate and adaptive immunity in the lung. Blood 100: 4193–4200. 12393686 10.1182/blood-2002-04-1102

[30] Car BD, Eng VM, Schnyder B, LeHir M, Shakhov AN, et al (1995) Role of interferon-gamma in interleukin 12-induced pathology in mice. Am J Pathol 147: 1693–1707. 7495294 PMC1869961

[31] Lapaque N, Walzer T, Meresse S, Vivier E, Trowsdale J (2009) Interactions between Human NK Cells and Macrophages in Response to Salmonella Infection. Journal of Immunology 182: 4339–4348. 10.4049/jimmunol.0803329 19299734

[32] Munder M, Mallo M, Eichmann K, Modolell M (1998) Murine macrophages secrete interferon gamma upon combined stimulation with interleukin (IL)-12 and IL-18: A novel pathway of autocrine macrophage activation. Journal of Experimental Medicine 187: 2103–2108. 9625771 10.1084/jem.187.12.2103 PMC2212367

[33] van der Velden AWM, Lindgren SW, Worley MJ, Heffron F (2000) Salmonella pathogenicity island 1-independent induction of apoptosis in infected macrophages by Salmonella enterica serotype typhimurium. Infection and Immunity 68: 5702–5709. 10992474 10.1128/iai.68.10.5702-5709.2000 PMC101526

[34] Kaiser P, Diard M, Stecher B, Hardt WD (2012) The streptomycin mouse model for Salmonella diarrhea: functional analysis of the microbiota, the pathogen's virulence factors, and the host's mucosal immune response. Immunological Reviews 245: 56–83. 22168414 10.1111/j.1600-065X.2011.01070.x

[35] Linden SK, Florin THJ, McGuckin MA (2008) Mucin Dynamics in Intestinal Bacterial Infection. Plos One 3. 10.1371/journal.pone.0003952 PMC2601037 19088856

[36] Bogaert S, De Vos M, Olievier K, Peeters H, Elewaut D, et al (2011) Involvement of endoplasmic reticulum stress in inflammatory bowel disease: a different implication for colonic and ileal disease? PLoS One 6: e25589. 22028783 10.1371/journal.pone.0025589 PMC3196494

[37] Heazlewood CK, Cook MC, Eri R, Price GR, Tauro SB, et al (2008) Aberrant mucin assembly in mice causes endoplasmic reticulum stress and spontaneous inflammation resembling ulcerative colitis. PLoS Med 5: e54. 18318598 10.1371/journal.pmed.0050054 PMC2270292

[38] Shkoda A, Ruiz PA, Daniel H, Kim SC, Rogler G, et al (2007) Interieukin-10 blocked endoplasmic reticulum stress in intestinal epithelial cells: Impact on chronic inflammation. Gastroenterology 132: 190–207. 17241871 10.1053/j.gastro.2006.10.030

[39] Soderholm JD, Perdue MH (2001) Stress and gastrointestinal tract. II. Stress and intestinal barrier function. Am J Physiol Gastrointest Liver Physiol 280: G7–G13. 11123192 10.1152/ajpgi.2001.280.1.G7

[40] Varki NM, Varki A (2007) Diversity in cell surface sialic acid presentations: implications for biology and disease. Laboratory Investigation 87: 851–857. 17632542 10.1038/labinvest.3700656 PMC7100186

[41] Smith WE, Kane AV, Campbell ST, Acheson DW, Cochran BH, et al (2003) Shiga toxin 1 triggers a ribotoxic stress response leading to p38 and JNK activation and induction of apoptosis in intestinal epithelial cells. Infect Immun 71: 1497–1504. 12595468 10.1128/IAI.71.3.1497-1504.2003 PMC148871

[42] Kaser A, Blumberg RS (2010) Endoplasmic reticulum stress and intestinal inflammation. Mucosal Immunol 3: 11–16. 19865077 10.1038/mi.2009.122 PMC4592136

[43] McGuckin MA, Linden SK, Sutton P, Florin TH (2011) Mucin dynamics and enteric pathogens. Nat Rev Microbiol 9: 265–278. 21407243 10.1038/nrmicro2538

[44] Songhet P, Barthel M, Stecher B, Muller AJ, Kremer M, et al (2011) Stromal IFN-gammaR-signaling modulates goblet cell function during Salmonella Typhimurium infection. PLoS One 6: e22459. 21829463 10.1371/journal.pone.0022459 PMC3145644

[45] Johansson MEV, Hansson GC (2011) Keeping Bacteria at a Distance. Science 334: 182–183. 21998374 10.1126/science.1213909

[46] Raouf AH, Tsai HH, Parker N, Hoffman J, Walker RJ, et al (1992) Sulphation of colonic and rectal mucin in inflammatory bowel disease: reduced sulphation of rectal mucus in ulcerative colitis. Clin Sci (Lond) 83: 623–626. 1335401 10.1042/cs0830623

[47] Mahdavi J, Sonden B, Hurtig M, Olfat FO, Forsberg L, et al (2002) Helicobacter pylori SabA adhesin in persistent infection and chronic inflammation. Science 297: 573–578. 12142529 10.1126/science.1069076 PMC2570540

[48] Endt K, Stecher B, Chaffron S, Slack E, Tchitchek N, et al (2010) The microbiota mediates pathogen clearance from the gut lumen after non-typhoidal Salmonella diarrhea. PLoS Pathog 6: e1001097. 20844578 10.1371/journal.ppat.1001097 PMC2936549

[49] Loetscher Y, Wieser A, Lengefeld J, Kaiser P, Schubert S, et al (2012) Salmonella transiently reside in luminal neutrophils in the inflamed gut. PLoS One 7: e34812. 22493718 10.1371/journal.pone.0034812 PMC3321032

[50] Winter SE, Winter MG, Xavier MN, Thiennimitr P, Poon V, et al (2013) Host-derived nitrate boosts growth of E. coli in the inflamed gut. Science 339: 708–711. 23393266 10.1126/science.1232467 PMC4004111

[51] Wohlgemuth S, Haller D, Blaut M, Loh G (2009) Reduced microbial diversity and high numbers of one single Escherichia coli strain in the intestine of colitic mice. Environ Microbiol 11: 1562–1571. 19245530 10.1111/j.1462-2920.2009.01883.x

[52] Moulin-Schouleur M, Schouler C, Tailliez P, Kao MR, Bree A, et al (2006) Common virulence factors and genetic relationships between O18:K1:H7 Escherichia coli isolates of human and avian origin. J Clin Microbiol 44: 3484–3492. 17021071 10.1128/JCM.00548-06 PMC1594794

[53] Kleessen B, Kroesen AJ, Buhr HJ, Blaut M (2002) Mucosal and invading bacteria in patients with inflammatory bowel disease compared with controls. Scand J Gastroenterol 37: 1034–1041. 12374228 10.1080/003655202320378220

[54] Malorny B, Anderson A, Huber I (2007) Salmonella real-time PCR-Nachweis. Journal Fur Verbraucherschutz Und Lebensmittelsicherheit-Journal of Consumer Protection and Food Safety 2: 149–156.

[55] Slezak K, Hanske L, Loh G, Blaut M (2013) Increased bacterial putrescine has no impact on gut morphology and physiology in gnotobiotic adolescent mice. Benef Microbes 4: 253–266. 23666100 10.3920/BM2012.0047

[56] Giannasca KT, Giannasca PJ, Neutra MR (1996) Adherence of Salmonella typhimurium to Caco-2 cells: Identification of a glycoconjugate receptor. Infection and Immunity 64: 135–145. 8557331 10.1128/iai.64.1.135-145.1996 PMC173738

[57] Severi E, Hood DW, Thomas GH (2007) Sialic acid utilization by bacterial pathogens. Microbiology-Sgm 153: 2817–2822. 10.1099/mic.0.2007/009480-0 17768226

[58] Clark MA, Reed KA, Lodge J, Stephen J, Hirst BH, et al (1996) Invasion of murine intestinal M cells by Salmonella typhimurium inv mutants severely deficient for invasion of cultured cells. Infection and Immunity 64: 4363–4368. 8926113 10.1128/iai.64.10.4363-4368.1996 PMC174381

[59] Foster N, Macpherson GG (2010) Murine Cecal Patch M Cells Transport Infectious Prions In Vivo. Journal of Infectious Diseases 202: 1916–1919. 21050122 10.1086/657415

