# Supplementary figures and images for: Commensal Akkermansia muciniphila Exacerbates Gut Inflammation in Salmonella Typhimurium-Infected Gnotobiotic Mice
Source: PLoS One. 2013 Sep 10;8(9):e74963. doi: 10.1371/journal.pone.0074963 (PMC3769299; doi:10.1371/journal.pone.0074963)

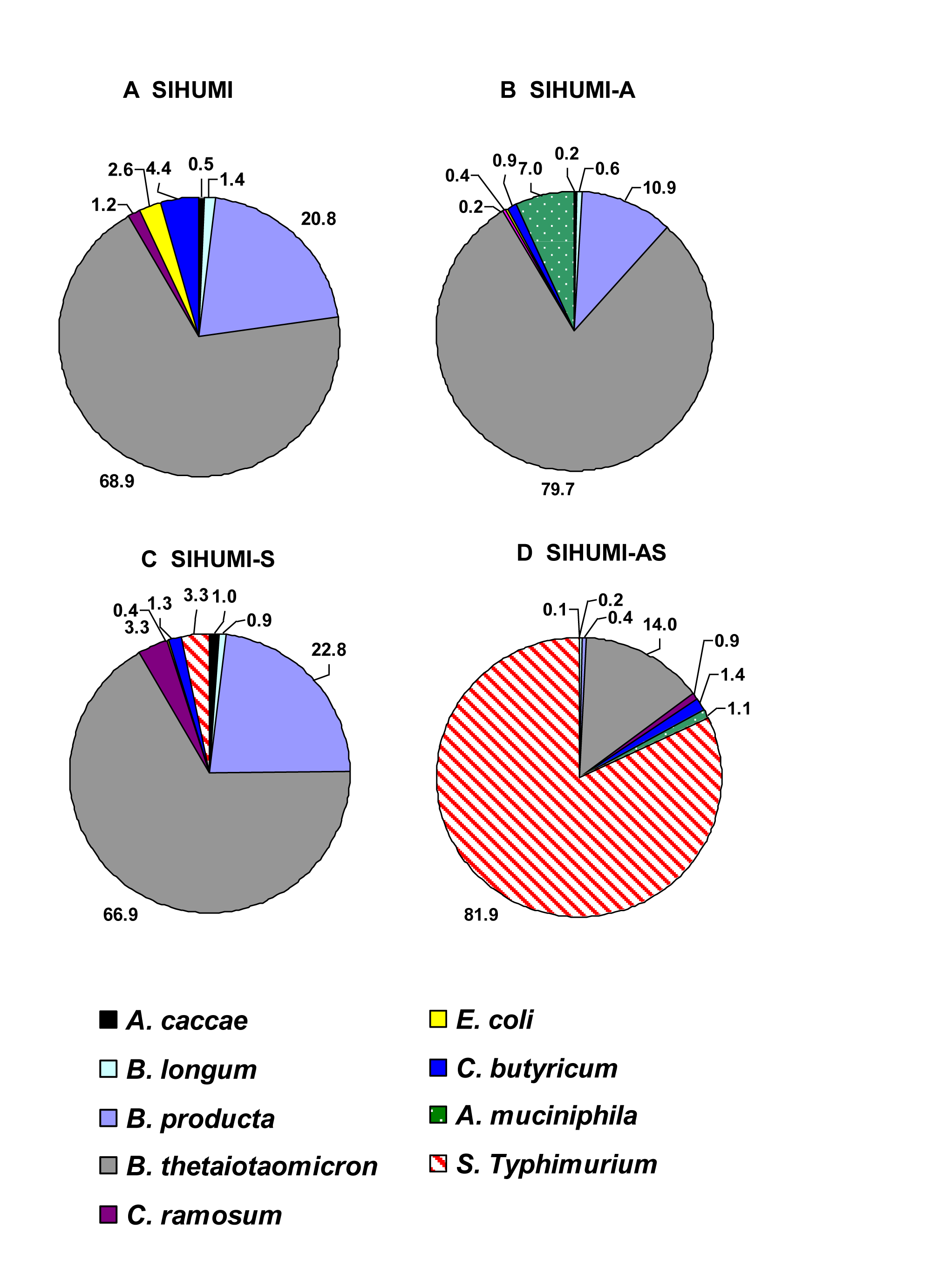

Supplement: Figure S1 — Presence of A. muciniphila renders S. Typhimurium the dominant species in colon of gnotobiotic SIHUMI mice. Colonic contents were recovered from gnotobiotic C3H mice assigned to 4 groups, differing in their microbial status: (A) Mice with a defined microbial community of eight bacterial species (SIHUMI), (B) SIHUMI mice colonized additionally with A. muciniphila (SIHUMI-A), (C) SIHUMI mice infected with S. (SIHUMI-S) and (D) SIHUMI mice colonized with A. muciniphila and 10 days later infected with S. Typhimurium (SIHUMI-AS). Total DNA was extracted and bacterial cell numbers were quantified by primers targeting the HSP60 gene of the SIHUMI members, the 16S rRNA gene of A. muciniphila and the ttr-region of S. Typhimurium using quantitative PCR. Calculation of the cell numbers was based on DNA obtained from cell suspensions containing known cell numbers of the targeted bacterial species (see materials and methods). Reduced proportion of SIHUMI members in SIHUMI-AS mice is attributed to an increase in the proportion of S. Typhimurium cells. Ten animals per group were used. The exact bacterial cell numbers and P-values for the differences between the groups are provided in Table S1. (TIF) [file pone.0074963.s001.tif]

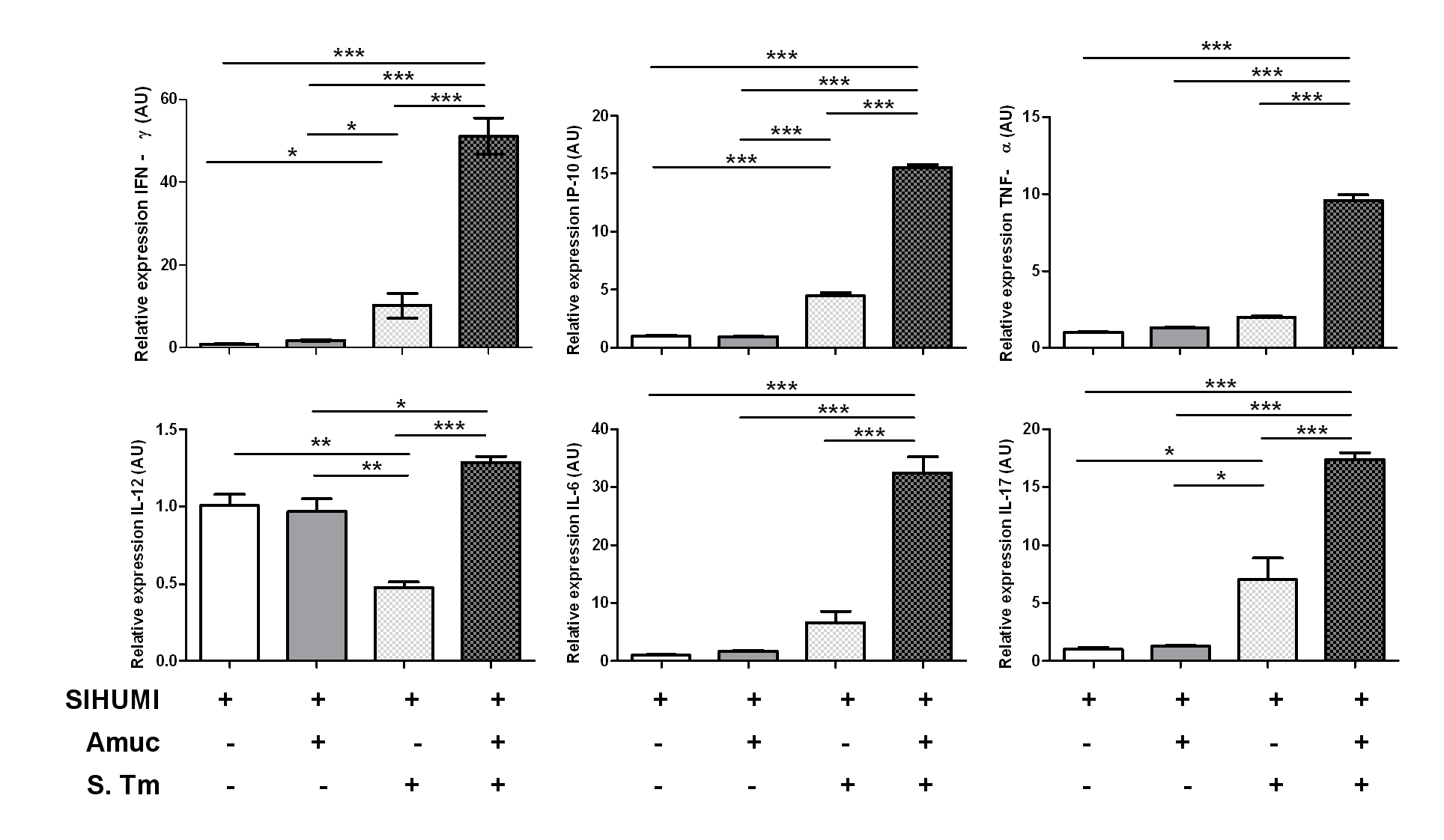

Supplement: Figure S2 — Presence of both A. muciniphila and S. Typhimurium is accompanied by increased colonic pro-inflammatory cytokine mRNA levels. Colonic mRNA levels of IFN-γ, IP-10, TNF-α, IL-12, IL-6 and IL-17 in gnotobiotic C3H mice were measured. mRNA was extracted from colon mucosa of mice belonging to either one of four groups: SIHUMI, SIHUMI-A, SIHUMI-S and SIHUMI-AS (see Figure. 1). The mRNA was converted to cDNA for quantitative real-time PCR measurement (see materials and methods). Inoculation of the gnotobiotic SIHUMI mice with A. muciniphila followed by S. Typhimurium infection (SIHUMI-AS) caused an increase in mRNA levels of pro-inflammatory cytokines. Data are expressed as mean±standard error. n = 6 per group. Star indicates statistically significant differences (*P<0.05, **P<0.01, ***P<0.001). AU: Arbitrary units. (Amuc: A. muciniphila; S. Tm: S. Typhimurium). (TIF) [file pone.0074963.s002.tif]

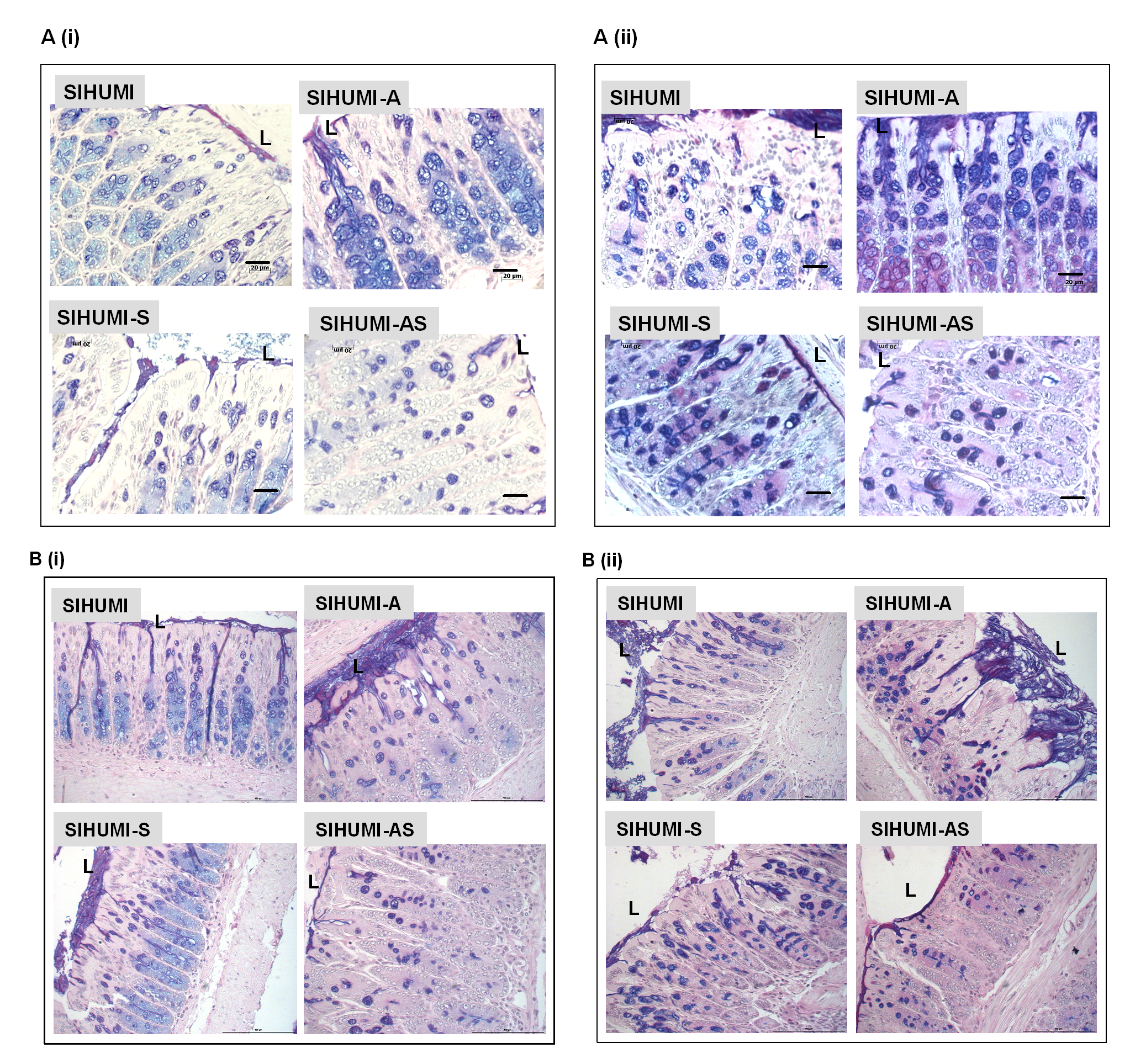

Supplement: Figure S3 — Presence of both A. muciniphila and S. Typhimurium caused reduction in number of mucin filled goblet cells in colon of SIHUMI mice. Carnoy-fixed cecal tissue sections (4 µm) from SIHUMI, SIHUMI-A, SIHUMI-S and SIHUMI-AS (see Figure. 1) mice were stained with periodic acid Schiff/Alcian blue (PAS/AB) at both pH 2.5 and pH 1.0. Images are representative of 5 mice per group. (A-i) All acidic mucins are stained blue with AB at pH 2.5 whereas all neutral mucins are stained magenta with PAS; (A-ii) highly sulphated mucins are stained blue with AB at pH-1. All the images from (A-i & A-ii) are obtained with a magnification of 1000-fold. Bars indicate 20 µm. (B-i) colonic tissues stained with PAS/AB at pH 2.5; (B-ii) colonic tissues stained with PAS/AB at pH 1.0 obtained with a magnification of 400-fold. Bars indicate 100 µm. SIHUMI-AS mice display the lowest number of positively stained colonic mucin-filled goblet cells compared to the other three groups at any given pH. L: lumen. (TIF) [file pone.0074963.s003.tif]

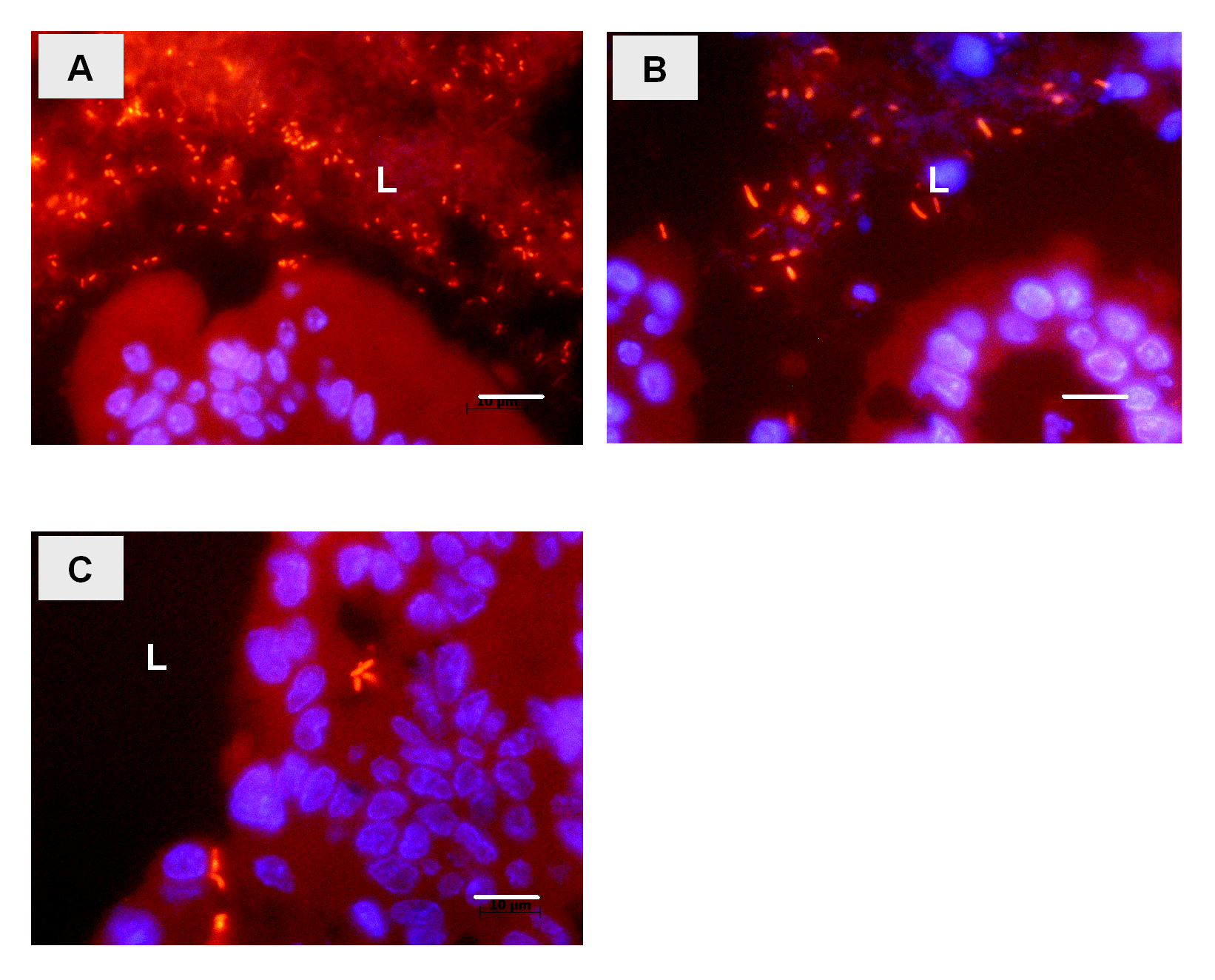

Supplement: Figure S4 — Detection of A. muciniphila and S. Typhimurium attached to mucosa in cecal tissue section by FISH. Thin sections (4 µm) of formalin fixed cecal tissue were used for the detection of A. muciniphila and S. Typhimurium by fluorescence in-situ hybridization (FISH) in gnotobiotic mice belonging to either one of the four groups: SIHUMI, SIHUMI-A, SIHUMI-S and SIHUMI-AS (see Figure. 1). Thin sections were hybridized with Cy3 labeled oligonucleotide probes (see Information S1) targeting A. muciniphila (S-S-MUC-1437-a-A-20) at 55°C and S. Typhimurium (L-S-Sal-1713-a-A-18) at 45°C. DNA was counterstained with 4',6-diamidino-2-phenylindole (DAPI). (A) A. muciniphila is in close contact to the epithelial surface in SIHUMI-A mice. (B) S. Typhimurium cells are found mostly on the epithelial cell surface of SIHUMI-S mice. (C) S. Typhimurium is in cecal tissue of SIHUMI-AS mice. Magnification 1000×. The scales represent 20 µm. L: lumen. (TIF) [file pone.0074963.s004.tif]
