# Supplementary material for: Commensal Akkermansia muciniphila Exacerbates Gut Inflammation in Salmonella Typhimurium-Infected Gnotobiotic Mice
Source: PLoS One. 2013 Sep 10;8(9):e74963. doi: 10.1371/journal.pone.0074963 (PMC3769299; doi:10.1371/journal.pone.0074963)
Supplement: Information S1 — Supporting Materials and Methods. (PDF) [file pone.0074963.s005.pdf]

**SUPPORTING INFORMATION (MATERIAL & METHODS):**

**S1: FISH analysis for bacterial detection in cecal tissue sections**

Formalin-fixed paraffin-embedded cecal tissue sections (4 µm) were initially treated with Roti-Histol (Carl Roth, Karlsruhe, Germany) and then hybridized at 50°C with the 5’-Cy3-labeled *A. muciniphila*-specific probe S-S-MUC-1437-a-A-20 (5’-CCTTGCGGTTGGCTTCAGAT-3’) [1] and at 45°C with the 5′-Cy3 labeled *Salmonella*-specific probe L-S-Sal-1713-a-A-18 (5’-AATCACTTCACCTACGTG-3’) (MWG Eurofins, Germany) as described previously [2]. After overnight hybridization sections were counter-stained with 4,6-diamidino-2-phenylindole (DAPI, Sigma-Aldrich) for visualization of cell nuclei and the slides were analyzed using a Nikon E600 epifluorescence microscope equipped with appropriate filter set, wavelength ranging from 330 to 700 nm (NIKON, Germany).

**S2: PAS/AB staining of colonic tissue samples**

Carnoy’s-fixed colonic tissue (Swiss-rolls) was sectioned at 4 µm and stained with periodic acid Schiff/Alcian blue (PAS/AB) at both pH 2.5 and 1 (at pH 2.5 acidic mucins are stained blue and neutral mucins are stained magenta, at pH 1: highly sulphated mucins are stained blue). Mucus layer thickness was measured on the colonic tissue sections. Approximately 30 crypts per section were analyzed using an Eclipse E600 microscope (NIKON, Germany) with Lucia G version 4.51 software for Windows 7 (Laboratory imaging s.r.o.). Images were captured with a MV-1500 digital camera (NIKON, Düsseldorf Germany).

1. Derrien M, Van Baarlen P, Hooiveld G, Norin E, Muller M, et al. (2011) Modulation of Mucosal Immune Response, Tolerance, and Proliferation in Mice Colonized by the Mucin-Degrader Akkermansia muciniphila. Front Microbiol 2: 166.
2. Nordentoft S, Christensen H, Wegener HC (1997) Evaluation of a fluorescence-labelled oligonucleotide tide probe targeting 23S rRNA for in situ detection of Salmonella serovars in paraffin-embedded tissue sections and their rapid identification in bacterial smears. Journal of Clinical Microbiology 35: 2642-2648.
