# Supplementary material for: Commensal Akkermansia muciniphila Exacerbates Gut Inflammation in Salmonella Typhimurium-Infected Gnotobiotic Mice
Source: PLoS One. 2013 Sep 10;8(9):e74963. doi: 10.1371/journal.pone.0074963 (PMC3769299; doi:10.1371/journal.pone.0074963)
Supplement: Table S1 — S. Typhimurium becomes the dominant species in colon of SIHUMI mice previously associated with A. muciniphila. (PDF) [file pone.0074963.s006.pdf]

Table S1. *S.* Typhimurium becomes the dominant species in colon of SIHUMI mice previously associated with *A. muciniphila*.

Data are expressed as mean ± standard error. Different superscripts indicate statistically significant differences (*$P \leq 0.05$).* n = 10 mice per group. DW: dry weight.

Colon	SIHUMI	SIHUMI-S	SIHUMI-A	SIHUMI-AS
	$\log_{10}$ (g^{-1} DW)	$\log_{10}$ (g^{-1} DW)	$\log_{10}$ (g^{-1} DW)	$\log_{10}$ (g^{-1} DW)
A caccae	8.6±0.6 ^{b}	9.2± 0.3 ^{b}	8.9±0.3 ^{b}	6.9±1.2 ^{a}
B longum	9.0±0.2 ^{b}	9.1±0.5 ^{b}	9.4±0.2 ^{b}	7.4±1.4 ^{a}
B producta	10.2±0.5 ^{b}	10.5±0.2 ^{b}	10.6±0.2 ^{b}	7.7±1.3 ^{a}
B thetaiotaomicron	10.7±0.5 ^{b}	11.0±0.3 ^{b}	11.5±0.4 ^{b}	9.2±0.9 ^{a}
C ramosum	9.0±0.3 ^{b}	9.7±0.3 ^{c}	8.9±0.5 ^{b}	8.0±1.1 ^{a}
E coli	9.3±0.4 ^{b}	8.7±0.3 ^{b}	9.2±0.6 ^{b}	6.1±0.8 ^{a}
C butyricum	9.5±0.3 ^{b}	9.3±0.4 ^{b}	9.5±0.3 ^{b}	8.2±1.4 ^{a}
A muciniphila	n.d.	n.d.	10.4±0.2 ^{b}	8.1±0.6 ^{a}
Salmonella	n.d.	9.7±0.2 ^{a}	n.d.	10.0±0.4 ^{a}
**Total bacteria**	10.6±0.7 ^{b}	10.7±0.6 ^{b}	11.1±0.8 ^{b}	9.3±0.7 ^{a}
